# Do engagement and behavioural mechanisms underpin the effectiveness of the Drink Less app?

**DOI:** 10.1038/s41746-024-01169-7

**Published:** 2024-06-29

**Authors:** Claire Garnett, Larisa-Maria Dinu, Melissa Oldham, Olga Perski, Gemma Loebenberg, Emma Beard, Colin Angus, Robyn Burton, Matt Field, Felix Greaves, Matthew Hickman, Eileen Kaner, Susan Michie, Marcus Munafò, Elena Pizzo, Jamie Brown

**Affiliations:** 1 Department of Behavioural Science and Health, University College London, London, UK; 2 School of Psychological Science, University of Bristol, Bristol, UK; 3 University of California, San Diego, San Diego, CA USA; 4 Tampere University, Tampere, Finland; 5 School of Health and Related Research, University of Sheffield, Sheffield, UK; 6 Addictions Directorate, Office for Health Improvement and Disparities, London, UK; 7 Institute of Psychiatry, Psychology and Neuroscience, King’s College London, London, UK; 8 Department of Psychology, University of Sheffield, Sheffield, UK; 9 Department of Primary Care and Public Health, Imperial College London, London, UK; 10 NICE (National Institute for Health and Care Excellence), London, UK; 11 Population Health Sciences, Bristol Medical School, University of Bristol, Bristol, UK; 12 Population Health Sciences Institute, Newcastle University, Newcastle upon Tyne, UK; 13 Centre for Behaviour Change, University College London, London, UK; 14 MRC Integrative Epidemiology Unit at the University of Bristol, Bristol, UK; 15 Department of Applied Health Research, University College London, London, UK

**Keywords:** Public health, Human behaviour

## Abstract

This is a process evaluation of a large UK-based randomised controlled trial (RCT) (*n* = 5602) evaluating the effectiveness of recommending an alcohol reduction app, Drink Less, compared with usual digital care in reducing alcohol consumption in increasing and higher risk drinkers. The aim was to understand whether participants’ engagement (‘self-reported adherence’) and behavioural characteristics were mechanisms of action underpinning the effectiveness of Drink Less. Self-reported adherence with both digital tools was over 70% (Drink Less: 78.0%, 95% CI = 77.6–78.4; usual digital care: 71.5%, 95% CI = 71.0–71.9). Self-reported adherence to the intervention (average causal mediation effect [ACME] = −0.250, 95% CI = −0.42, −0.11) and self-monitoring behaviour (ACME = −0.235, 95% CI = −0.44, −0.03) both partially mediated the effect of the intervention (versus comparator) on alcohol reduction. Following the recommendation (self-reported adherence) and the tracking (self-monitoring behaviour) feature of the Drink Less app appear to be important mechanisms of action for alcohol reduction among increasing and higher risk drinkers.

## Introduction

Reducing increasing and higher risk alcohol consumption (defined as scoring 8 or more on the Alcohol Use Disorders Identification Test [AUDIT]) is a public health priority^1^. Digital interventions, such as websites and smartphone applications (‘apps’), potentially have a broad reach and low incremental costs for delivering alcohol interventions at scale^2^. Apps are a particularly promising mode of intervention delivery because smartphones have become increasingly affordable to end users, with approximately 84% of the UK population having access to a smartphone^3^. Meta-analyses have shown there is evidence for the effectiveness of digital interventions at reducing alcohol consumption^4,5^. Nonetheless, most of the interventions included in these meta-analyses were websites rather than apps. Furthermore, despite many alcohol reduction apps being available on app stores in the United Kingdom (UK), none have been evaluated in a randomised controlled trial (RCT) among the adult general population. To address this gap, we conducted an RCT (the iDEAS trial; iOS Drink Less, evaluating the Effectiveness of an Alcohol Smartphone app) to evaluate the effectiveness of recommending the evidence- and theory-informed app, Drink Less, in reducing alcohol consumption among increasing and higher risk drinkers in the UK, compared with usual digital care^6,7^. After accounting for missing data using multiple imputation, we found a two-unit reduction (95% CI = −3.76 to −0.24) in weekly alcohol consumption among the Drink Less group after 6 months compared with usual digital care, though the effect without imputation (where non-responders were assumed to be drinking at baseline levels) was weaker (−0.98 units, 95% CI = −2.67 to 0.70)^7^.

**Fig. 1:**
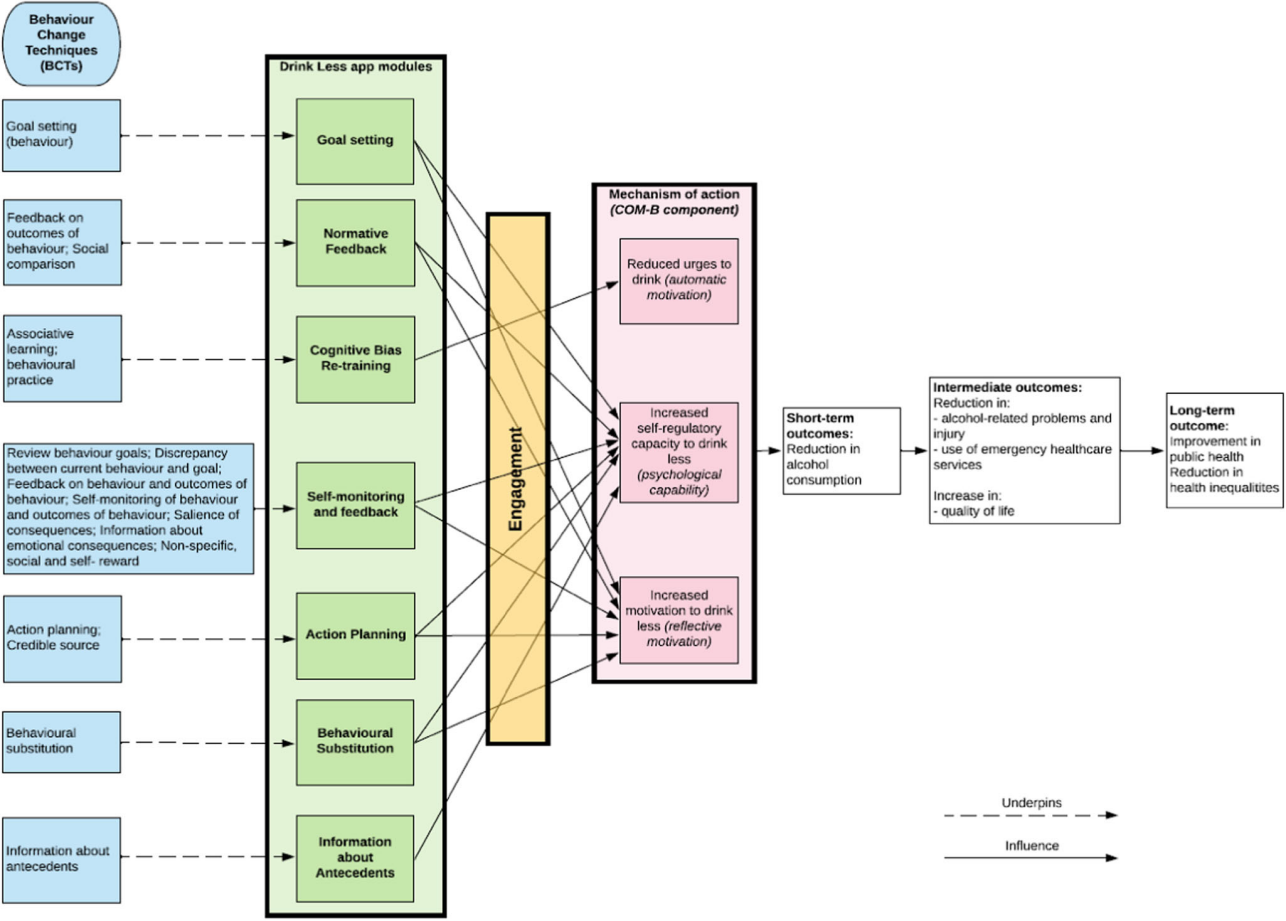
Logic model of the Drink Less app. Logic model showing which behaviour change techniques map onto the evidence-based Drink Less app modules and the proposed mechanisms of action: engagement with the interventional components of the Drink Less app, which, in turn, influences participants’ behavioural characteristics, including urges to drink, motivation to drink less and self-regulatory behaviour, leading to short-term, intermediate and long-term outcomes.

In addition to establishing whether an alcohol reduction app is effective, it is critical to understand its mechanisms of action, or, in other words, why it is effective. Understanding the underlying processes through which an intervention has its effects can help design more effective interventions^8^. To this end, process evaluations can help test hypothesised causal pathways using quantitative data^9^. In the current study, the overarching theoretical framework underpinning the Drink Less app is the COM-B model of behaviour^10^, and the proposed mechanisms of action were engagement with the interventional components of the Drink Less app (see ‘Methods’ section)^11,12^, which, in turn, influences participants’ behavioural characteristics, including urges to drink, motivation to drink less and self-regulatory behaviour (see Fig. 1 for the logic model).

This study used data from the iDEAS trial comparing the effectiveness of the Drink Less app with usual digital care in reducing alcohol consumption in increasing and higher risk drinkers, focusing on participants’ behavioural characteristics and engagement with the intervention as part of the embedded mixed-methods process evaluation. The qualitative component evaluating the acceptability of the digital tools is reported elsewhere^13^ and found that Drink Less was perceived as being ethical, easy, user-friendly and effective. The following research questions were addressed:

1. To what extent do participants self-report adhering to their recommended digital tool and how does this differ by group?
2. Among participants in the intervention group, to what extent do participants engage with the Drink Less app in terms of (i) downloading the app; (ii) depth; (iii) frequency; (iv) duration; and (v) amount of use over the 6-month period from the date of recommendation?
3. Does motivation to drink less at baseline moderate the effect of the intervention on alcohol reduction in increasing and higher risk drinkers at 6-month follow-up?
4. Do (i) urges to drink, self-regulatory and self-monitoring behaviour at 6-month follow-up, and (ii) self-reported adherence at 1- or 6-month follow-up to the recommended digital tool mediate the effect of the intervention on alcohol reduction in increasing and higher risk drinkers at 6-month follow-up?
5. Among participants in the intervention group, does extent of behavioural engagement with the Drink Less app mediate the effect of self-reported adherence on intervention effectiveness on alcohol reduction in increasing and higher risk drinkers at 6-month follow-up?

## Results

### RQ1: Participant self-reported adherence to recommended digital tool

Among the intervention group, 78.0% (95% CI = 77.6–78.4) self-reported adherence (at either 1- or 6-month follow-up) to their digital tool, which was significantly greater than the 71.5% (95% CI = 71.0–71.9) in the comparator group (*t* = 18.470, *p* = 0.034), see Table 1. The pattern of results was similar when conducting a complete case analysis (see Table 1).

**Table 1:** Self-reported adherence to their recommended digital tool by group

	1-month follow-up	6-month follow-up	1- or 6-month follow-up
Multiple imputation for missing data (*n* = 5602)			
Intervention (Drink Less), *n* = 2788	72.0% (95% CI = 71.6–72.4)	67.4% (95% CI = 67.0–67.9)	78.0% (95% CI = 77.6–78.4)
Comparator (NHS alcohol advice webpage), *n* = 2814	64.3% (95% CI = 63.8–64.7)	57.0% (95% CI = 56.5–57.5)	71.5% (95% CI = 71.0–71.9)
Complete case analysis			
Intervention (Drink Less)	79.9% (*n* = 1435), 95% CI = 77.9–81.7	69.3% (*n* = 1481), 95% CI = 67.3–71.2	78.8% (*n* = 1782), 95% CI = 77.0–80.4
Comparator (NHS alcohol advice webpage)	71.2% (*n* = 1336), 95% CI = 69.1–73.2	56.3% (*n* = 1236), 95% CI = 54.2–58.4	70.9% (*n* = 1662), 95% CI = 69.0–72.7

### RQ2: Extent of behavioural engagement with Drink Less among intervention group

Among participants in the intervention group, 1858 participants (66.6%, 95% CI = 64.9–68.4%) downloaded the Drink Less app and entered their email address. Ten participants downloaded the app outside of the 6-month period and were excluded.

Of the 1858, 128 participants downloaded the app multiple times (109 participants downloaded the app twice, 15 downloaded it three times, 3 downloaded it four times and 1 participant downloaded it five times). The median length of time for participants to download the app from being recommended it in the baseline survey was 3 min (mean = 1 day).

Among all participants in the intervention group (*n* = 2788), i.e., including the 33% of participants who did not download the app at all, they had a mean number of 34 sessions, spent a mean of 54 min on the app, used it for a mean of 25 days and viewed a mean of 17 unique screens, see Table 2. The figures were higher among only those who downloaded the app.

**Table 2:** Engagement data for participants in the intervention group

Engagement	All participants in intervention group (*n* = 2788)	Participants who downloaded Drink Less (*n* = 1858)
Number of sessions (frequency)		
Mean (SD)	34.3 (65.06)	51.4 (73.96)
Median (IQR)	5 (0, 32)	16 (5, 67)
Time on app in minutes (amount)		
Mean (SD)	54.0 (115.25)	81.1 (133.20)
Median (IQR)	10 (0, 54)	32 (10, 95)
Number of days used (duration)		
Mean (SD)	25.3 (44.44)	38.0 (49.83)
Median (IQR)	4 (0, 26)	13 (4, 52)
Number of unique screens viewed (depth)		
Mean (SD)	17.4 (14.94)	26.1 (10.40)
Median (IQR)	18 (0, 30)	26 (18, 34)

*SD* standard deviation, *IQR* interquartile range.

### RQ3: Motivation to drink less as a moderator of intervention effectiveness

No significant interaction was detected between participants’ motivation to drink less at baseline and their group allocation (intervention versus comparator) on intervention effectiveness (*F*_1,5011.39_ = 0.285, *p* = 0.594; log-transformed coefficient = 0.741, 95% CI = −1.927 to 3.410). This indicates that there was insufficient evidence to support a moderating effect of motivation to drink less at baseline having a differential effect on alcohol reduction in increasing and higher risk drinkers at 6-month follow-up (using multiple imputation for missing outcome data at 6-month follow-up).

The same pattern of results was found in the sensitivity analysis (when using a complete case approach at 6-month follow-up) with no significant interaction detected between motivation to drink less and group allocation on the primary outcome (*F*_1,3628546.53_ = 0.023, *p* = 0.590; log-transformed coefficient = 0.751, 95% CI = −1.979 to 3.481).

### RQ4: Urges to drink, self-regulatory and self-monitoring behaviour and self-reported adherence as mediators of intervention effectiveness

Among all participants, self-reported adherence to the recommended digital tool partially mediated the effect of the intervention on alcohol reduction in increasing and higher risk drinkers at 6-month follow-up (average causal mediation effects [ACME] = −0.250, 95% CI = −0.42 to −0.11) and there was a direct effect of intervention group on alcohol reduction when not considering the path of self-reported adherence as a mediator (average direct effects [ADE] = −1.966, 95% CI = −3.68 to −0.13).

Self-monitoring behaviour partially mediated the effect of the intervention group on alcohol reduction in increasing and higher risk drinkers at 6-month follow-up (ACME = −0.235, 95% CI = −0.44 to −0.03) and there was a direct effect of intervention group on alcohol reduction when not considering self-monitoring behaviour as a mediator (ADE = −1.966, 95% CI = −3.65 to −0.12).

No mediation of urges to drink (ACME = −0.096, 95% CI = −0.48 to 0.33) or self-regulatory behaviour (ACME = 0.142, 95% CI = −0.28 to 0.60) was detected.

In the sensitivity analysis among only those participants who completed the 6-month follow-up, both mediation results persisted but in both cases there was no significant direct effect of intervention group on alcohol reduction when not considering the mediators (see Supplementary Table 8).

### RQ5: Extent of behavioural engagement as a mediator of the effect of self-reported adherence on intervention effectiveness in the intervention group

Among all participants in the intervention group (*n* = 2788), with treatment status as self-reported adherence to the app, no causally mediating effects of number of sessions (ACME = −0.009, 95% CI = −0.10 to 0.07), time on app (ACME = −0.017, 95% CI = −0.12 to 0.06), number of days used (ACME = −0.009, 95% CI = −0.10 to 0.06), or unique screens viewed (ACME = −0.024, 95% CI = −0.15 to 0.06) on alcohol reduction at 6-month follow-up were detected. This indicates that there was no causal mediation effect on alcohol reduction among the treatment status (i.e., those who self-reported adherence to the app) as a result of the mediator (i.e., extent of behavioural engagement). Among all participants in the intervention group, no direct effects of the treatment status (i.e., self-reported adherence to the app) on alcohol reduction at 6-month follow-up were detected, when not considering the engagement measures as mediators (number of sessions, ADE = 0.386, 95% CI = −2.49 to 3.31; time on app, ADE = 0.468, 95% CI = −2.55 to 3.56; number of days used, ADE = 0.413, 95% CI = −2.42 to 3.34; unique screens viewed, ADE = 0.523, 95% CI = −2.54 to 3.57).

In the sensitivity analysis among only the participants in the intervention group who completed the 6-month follow-up, no causally mediating effects were detected (see Supplementary Table 9). However, there were direct effects of the treatment status (i.e., self-reported adherence to the app) on alcohol reduction at 6-month follow-up, when not considering the behavioural engagement measures as mediators (see Supplementary Table 9). This indicates that while there were no causal mediation effects on alcohol reduction among the treatment status (i.e., those who self-reported adherence to the app) as a result of the engagement measures as a mediator, there was a direct effect of self-reported adherence to the app on alcohol reduction.

## Discussion

This process evaluation investigated engagement with an alcohol reduction app, Drink Less, and participants’ behavioural characteristics as potential mechanisms of action underlying the effectiveness of the app. Self-reported adherence to both digital tools was over 70% and was significantly higher for Drink Less than for usual digital care. Self-reported adherence to the intervention and self-reported self-monitoring behaviour both partially mediated the effect of Drink Less, compared with usual digital care, on alcohol reduction.

Participants’ self-reported adherence was 78% for Drink Less and 72% for the NHS alcohol advice webpage. While 78% of participants in the intervention group self-reported using Drink Less, app data showed that only 67% downloaded the app. App downloads is a behavioural measure of adherence; however, it may be that some participants downloaded the app but did not enter their email address on the onboarding page, meaning that their app data could not be linked with their participation in the trial.

The extent of behavioural engagement with the Drink Less app among all participants randomised to the intervention (i.e., including the 33% of participants who did not download the app) was also investigated. Participants had a mean number of 34 sessions, spent a mean of 54 min on the app, used it for a mean of 25 days and viewed a mean of 17 unique screens. This data was positively skewed with participants using the app a median number of 5 sessions, 10 min, 4 days and viewing 18 unique screens. This suggests that there was a smaller group of ‘super users’ who were engaging intensively with the app and we are planning future research to characterise this group. The presence of highly engaged users has been found for other digital interventions^14^. When excluding participants who did not download the app, the mean number of 51 sessions, 81 min on the app, used it for 38 days and viewed 26 unique screens. While there are currently no guidelines for what constitutes adequate engagement, the data reported here suggest relatively high engagement with the Drink Less app when compared with engagement metrics reported in other studies of mobile health apps^15^. Comparing directly with a previous study of 672 Drink Less users in 2016, over a one-month period, people who downloaded the app had a median number of 5 sessions, 17 min on the app and used it for 4 days^16^. The current study measured engagement over a 6-month period and while the median values for users of the app were higher (median sessions = 16; median time = 32 min; median days used = 13) they were not six times higher. This could be due to the way in which participants found the app: in the 2016 study, users found the app in the iTunes store while in the current study participants were recommended (but did not choose themselves) to use the app (the recommendation was made remotely at the end of the baseline survey). Alternatively, it could relate to engagement tending to be higher in the earlier weeks and months and tailing off over time^17^.

Participants self-reported adherence to the intervention and self-reported self-monitoring behaviour (i.e., how often they kept track of how many units of alcohol they drank each week) both partially mediated their alcohol reduction at 6-month follow-up. This aligns with a previous investigation of behavioural engagement in a large sample of Drink Less users (over 19,000) which found that 85% of screen views occurred in the Self-monitoring & Feedback module of the app^17^ suggesting that this app module is how most users engage with the app. Furthermore, recent systematic reviews of mobile health apps have reported positive associations between self-monitoring behaviour and higher user engagement^18^, and self-monitoring behaviour and behaviour change^19^. These findings suggest that following the recommendation to use a digital intervention and tracking alcohol consumption are mechanisms which underlie the effectiveness of the Drink Less app.

There was no evidence that baseline motivation to drink less had a moderating effect of the intervention on alcohol reduction in increasing and higher risk drinkers at 6-month follow-up. This could be partially explained by a ceiling effect: the trial sample consisted of participants who were all motivated to cut down on alcohol reduction, which was reflected by the large reduction in alcohol consumption that was seen across both groups in the trial^7^. We found no evidence that self-regulatory behaviour or urges to drink had a mediating effect of the intervention on alcohol reduction, though there were reductions in both how difficult participants found it to control their drinking and in their strength of urges to drink from baseline to 6-month follow-up across both groups. The relationship between self-regulatory behaviour and alcohol reduction in the literature is mixed, with some research indicating that self-regulatory behaviour is related to alcohol-related consequences and not with alcohol consumption per se^20,21^. Furthermore, a contextual model of self-regulation change mechanisms in individuals with addictive disorders has been proposed, emphasising that the role of self-regulatory behaviour as a mechanism of behaviour change might depend on individual contextual factors^22^. The lack of evidence for self-regulatory behaviour mediating the effect of the intervention on alcohol reduction could be related to the fact that contextual factors were not specifically assessed in the iDEAS trial. However, research focusing on the importance of context in alcohol consumption is currently underway^23^.

Among participants in the intervention group (for whom we had data on their behavioural engagement), the effect of self-reported adherence on alcohol reduction did not appear to be mediated by the extent of behavioural engagement with the intervention (amount, duration, frequency or amount). This suggests that downloading the Drink Less app and following the recommendation is the critical engagement mechanism of the app’s effectiveness. That we did not detect a mediation effect of behavioural engagement with the app leading to better outcomes could be because behavioural engagement extends over time (e.g., frequency is an aggregated indicator, over time) and there might be dynamic feedback loops that were not accounted for here. It is also possible that any individual effect related to the behavioural engagement measures recorded within the app were overridden by the direct effect of self-reported adherence on alcohol reduction. We are planning future research to conduct more detailed modelling on exactly how users engage with the app in terms of which components, in what order and for how long to try and unpick further how engagement may relate to effectiveness.

This study reports on the findings from an embedded process evaluation of a large RCT of a theory- and evidence-based alcohol reduction app, Drink Less. We identified links between the outcome measures and participant engagement with the intervention and potential behavioural mechanisms of action.

Nonetheless, there are limitations. Firstly, given that self-reported adherence to the app was higher than the objective number of downloads, this analysis may have missed some participants who downloaded the app but failed to provide linkage data. Secondly, it was a strong assumption that self-reported adherence and engagement measures only relate to the pre-treatment covariates given that engagement is dynamic and fluctuates in response to time-varying factors^24^. Furthermore, we assumed each mediator was independent of each other, which they may not be, and it might be that engagement leads to changes to the behavioural mechanisms of actions, which then led to alcohol reduction. Using dynamic structural equation modelling could enable examination of multiple independent variables, mediators or outcomes^25,26^ and could be considered in future research. Another limitation was the reliance on self-report data for adherence and alcohol consumption. The self-reported adherence was higher than the objective app downloads though this may be due to the limitations in the ability to automatically link app usage with trial data, with some users potentially not including their email address or a different one to that used in the trial. However, the experimental design and remote recruitment with minimal research contact means there is likely limited differential bias between the two groups in the self-report data.

Finally, we were unable to assess whether behavioural engagement measures mediated the effect of the intervention on alcohol reduction as we were unable to measure behavioural engagement in the comparator group for the NHS alcohol advice webpage. As a result, we could only test whether engagement was a mediator among the intervention group, and whether engagement mediated the effect of self-reported adherence on alcohol reduction. However, it may have been that any individual mediation effect due to engagement was obscured by the direct effect of the self-reported adherence on alcohol reduction.

This study showed that engagement with the recommended digital tools was high in a large sample of digitally literate and motivated increasing and higher risk drinkers, with over 70% self-reporting adherence to either the Drink Less app or the NHS alcohol advice webpage. Self-reported adherence to the recommended digital tool partially mediated the effect of the intervention on alcohol reduction at 6-month follow-up indicating the importance of following the recommendation. Self-monitoring behaviour, i.e., how often users tracked their drinking, partially mediated the effect of the intervention on alcohol reduction suggesting that the Self-monitoring & Feedback module of the Drink Less app appears to be an important mechanism of action.

## Methods

### Ethics

Ethics approval was granted by the ethics committee at University College London (16799/001) and the trial was registered on the ISRCTN registry for clinical trials (ISRCTN64052601).

### Pre-registered protocol

The pre-registered study protocol can be found on the Open Science Framework: https://osf.io/2s7ft. Two changes were made to the pre-registered study protocol. Namely, the sensitivity analysis for research question 5 to test for the possible existence of unobserved pre-treatment covariates using the *medsens* function in R was not conducted, as this function could not be used on multiply imputed datasets. Furthermore, three descriptive research questions were dropped from the main manuscript, but all are reported in Supplementary File 1.

### Design

This was a process evaluation that assessed engagement and mechanisms of action of an intervention, the Drink Less app, embedded within a larger RCT^6^. Findings pertaining to the intervention acceptability are reported elsewhere^7^.

### Setting and sample

As described elsewhere^6^, participants from the UK were invited to take part in a trial evaluating the long-term effectiveness and cost-effectiveness of the digital recommendation of the Drink Less app, compared with advice from the National Health Service (NHS) alcohol advice webpage (usual digital care), in reducing alcohol consumption. Participants were recruited between July 2020 and March 2022 and had to be aged 18+, increasing and higher risk drinkers (AUDIT score ≥8), live in the UK, have access to an iOS device and want to drink less alcohol.

### Digital tools

Drink Less is a stand-alone app-based intervention that is freely available via the Apple app store in the UK^27^. Drink Less was developed for increasing and higher risk drinkers to help them reduce their alcohol consumption. Drink Less consists of evidence-based modules to help users change their drinking behaviour: Goal Setting, setting weekly ‘drinking reduction’ goals; Self-monitoring & Feedback, monitoring alcohol consumptions and seeing progress on goals; Action Planning, creating plans for dealing with difficult drinking situations; Normative Feedback, providing personalised feedback on how an individual’s drinking behaviour compares to the norm; Cognitive Bias Re-training, a game for retraining users’ automatic biases for alcoholic drinks; Behavioural Substitution, planning to substitute drinking with a neutral behaviour; and Information about Antecedents, providing users with information about situations and events, emotions and cognitions that predict their drinking. These evidence-based modules map to behaviour change techniques (see Fig. 1). The development, refinement, and content of the original Drink Less version is reported in full elsewhere^11,12^.

There were no specific requirements for participants when using the Drink Less app. On downloading Drink Less, users are asked to complete the AUDIT, provide sociodemographic details and then receive the Normative Feedback. Users are then guided through Goal Setting and shown how the key features of the app work before arriving on the landing page of the app with suggestions for the user to complete each day. The app provides a toolbox of features for users to choose from as and when they want. The app is not tailored to the user except for personalised feedback in two modules: Normative Feedback and Self-Monitoring & Feedback.

The comparator group received the recommendation to view the NHS alcohol advice webpage on ‘Tips on cutting down’^28^. This can be considered reflective of ‘usual digital care’ in this context as it is the digital support currently available to treatment-seeking individuals from the NHS.

### Measures

Participants self-enrolled into the study and responded to a web-based screening questionnaire, which assessed the inclusion and exclusion criteria, including the full AUDIT. Informed consent was obtained from all eligible participants. The baseline and follow-up surveys (measuring alcohol consumption, sociodemographic characteristics, self-reported adherence and behavioural characteristics) were conducted online using Qualtrics.

Self-reported weekly alcohol consumption was measured at baseline and at the 6-month follow-up, calculated using the 3-item Alcohol Use Disorders Identification Test—Consumption (AUDIT-C)^29^. The AUDIT-C asks about frequency (‘How often do you have a drink containing alcohol’ with five response options: Never; Monthly or less; 2 to 4 times per month; 2 to 3 times per month; 4 times of more per month), quantity (‘How many units of alcohol do you drink on a typical day when you are drinking?’ with five response options: 0 to 2; 3 to 4; 5 to 6; 7 to 9; 10 or more) and frequency of heavy episodic drinking (‘How often have you had 6 or more units on a single occasion in the last year?’ with five response options: Never; Less than monthly; Monthly; Weekly; Daily or almost daily).

Sociodemographic characteristics were recorded at baseline. Participants were asked to report their age (in years), gender (% female), ethnicity (% white), education (% post-16 education qualifications), occupation (to derive social grade ABC1: managerial, professional and intermediate occupations versus C2DE: skilled, semi-skilled, unskilled manual and lowest grade worked or unemployed) and income level (% > £26,000).

Self-reported adherence was measured at the 1-and 6-month follow-up surveys for all participants. It asked ‘Did you look at or use the digital tool we recommended? It doesn’t matter either way, you will still be paid, but it will help us draw more accurate conclusions if you answer honestly.’ with Yes/No response options. It is an alternative measure of engagement for cases where it is not possible to automatically measure engagement (e.g., for participants randomised to the comparator).

Behavioural engagement can be automatically measured through app usage logs for individuals randomised to the intervention over a 6-month period from the date of the recommendation (i.e., when the iDEAS trial baseline survey was completed). Engagement with digital interventions can be defined as ‘the extent of digital behaviour change intervention use (e.g., frequency, amount, duration, depth)’^30^. Frequency of engagement was assessed by number of sessions, where a new session was defined as a new screen view after 30 min of inactivity^31^. Amount of engagement was assessed by time on app, in minutes. Duration of engagement was assessed by the number of days the app was used. Depth of engagement was assessed by the number of available screens viewed (without considering time spent viewing each screen). While not fine-grained, these measures provide more objective information on how participants interact with the app, to supplement data on self-reported adherence of the intervention. The number of participants who downloaded the app multiple times is also reported. If a participant downloaded the app multiple times (and entered their email address each time), their total engagement over the multiple instances was summed. For any participant who did not download Drink Less, the app download was recorded as ‘no’ and the engagement measures were recorded as 0.

Behavioural characteristics measures were assessed as potential mechanisms of action in the baseline and 6-month follow-up surveys using four measures for all participants: urges to drink; motivation to drink less; self-regulatory behaviours (how difficult to control drinking) and self-monitoring behaviours (how often alcohol units are tracked) (see Fig. 1 for the Logic Model). Strength of urges to drink was measured with the question ‘How strongly have you felt the urge to drink alcohol in the past 24 hours?’ with a 6-point scale for responses: not at all; slight; moderate; strong; very strong; extremely strong. Motivation to drink less was measured with the Motivation to Stop Scale^32,33^ where participants were asked: ‘Which of the following describes you?’ with the following options: (1) I REALLY want to cut down on drinking alcohol and intend to in the next month; (2) I REALLY want to cut down on drinking alcohol and intend to in the next 3 months; (3) I want to cut down on drinking alcohol and hope to soon; (4) I REALLY want to cut down on drinking alcohol but I don’t know when I will; (5) I want to cut down on drinking alcohol but haven’t thought about when; (6) I think I should cut down on drinking alcohol but don’t really want to; (7) I don’t want to cut down on drinking alcohol. Self-regulatory behaviour was measured with the question ‘How difficult do you find it to control your drinking?’ with a 5-point scale for responses: not at all; slightly; moderately; very; extremely^34^. Self-monitoring behaviour was measured with the question ‘How often, if at all, do you keep track of how many units of alcohol you personally drink each week?’ with a 5-point scale for responses: never; rarely; sometimes; very often; always.

### Statistical analyses

All statistical analyses were conducted in R Studio (v2023.06). Multiple imputation (R package: Amelia) using baseline characteristics (gender, ethnicity, education, occupation, age and income) using 20 imputed datasets combined using Rubin’s rules was used for self-reported adherence, behavioural characteristics and alcohol reduction for the 20% of trial participants who did not respond to the 6-month follow-up survey^35,36^. Sensitivity analyses were also conducted using only the data from those participants responding to 6-month follow-up. Mediators of interest were assessed for skewness and log-transformed if necessary: motivation to drink less at baseline was negatively skewed and log-transformed, and all behavioural engagement measures were positively skewed and log-transformed (after adding 1 to avoid issues of infinity when log-transforming). For descriptive statistics, medians and interquartile ranges were reported to account for any potential skewness in the data.

For RQ1 (participant self-reported adherence to the recommended digital tool), among all participants, the proportion and 95% confidence interval (CI) of participants in each group self-reporting using their recommended digital tool (intervention or comparator) at (1) 1-month follow-up, (2) 6-month follow-up and (3) 1- or 6-month follow-up are reported.

For RQ2 (extent of behavioural engagement with Drink Less among intervention group), among participants in the intervention group (i.e., recommended to download the *Drink Less* app), the proportion and 95% CI of participants who downloaded the *Drink Less* app (i.e., input their email address when requested) is reported. Furthermore, the above analysis is repeated as a secondary analysis with those participants who did not download the app excluded. The length of time for participants to download the app from being recommended is also reported.

**Fig. 2:**
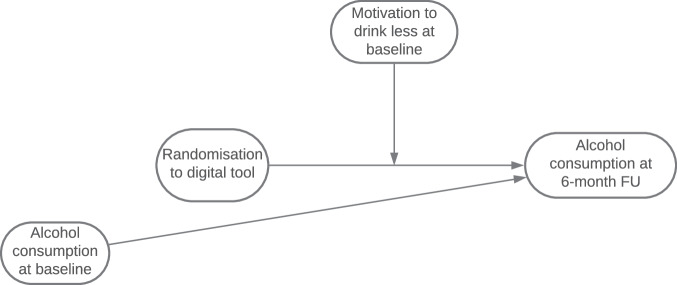
Moderation analysis for motivation to drink less. Motivation to drink less as a moderator of intervention effectiveness was assessed by including an interaction term between the moderator of interest (motivation to drink less at baseline, log transformed due to negative skewed data) and group allocation on the primary outcome in the primary analysis model from the main trial paper (a one-way ANCOVA examining the effect of group allocation on the primary outcome, weekly alcohol consumption at 6-month follow-up, adjusting for baseline consumption using multiple imputation for missing outcome data at 6-months).

For RQ3 (motivation to drink less as a moderator of intervention effectiveness), the main analysis model from the main trial paper is reported (a one-way ANCOVA examining the effect of group allocation on the primary outcome, weekly alcohol consumption at 6-month follow-up, adjusting for baseline consumption using multiple imputation for missing outcome data at 6-months), including an interaction term between the moderator of interest (motivation to drink less at baseline, log transformed due to negative skewed data) and group allocation on the primary outcome (using multiple imputation for missing data at 6-month follow-up), adjusting for alcohol consumption at baseline, see Fig. 2.

**Fig. 3:**
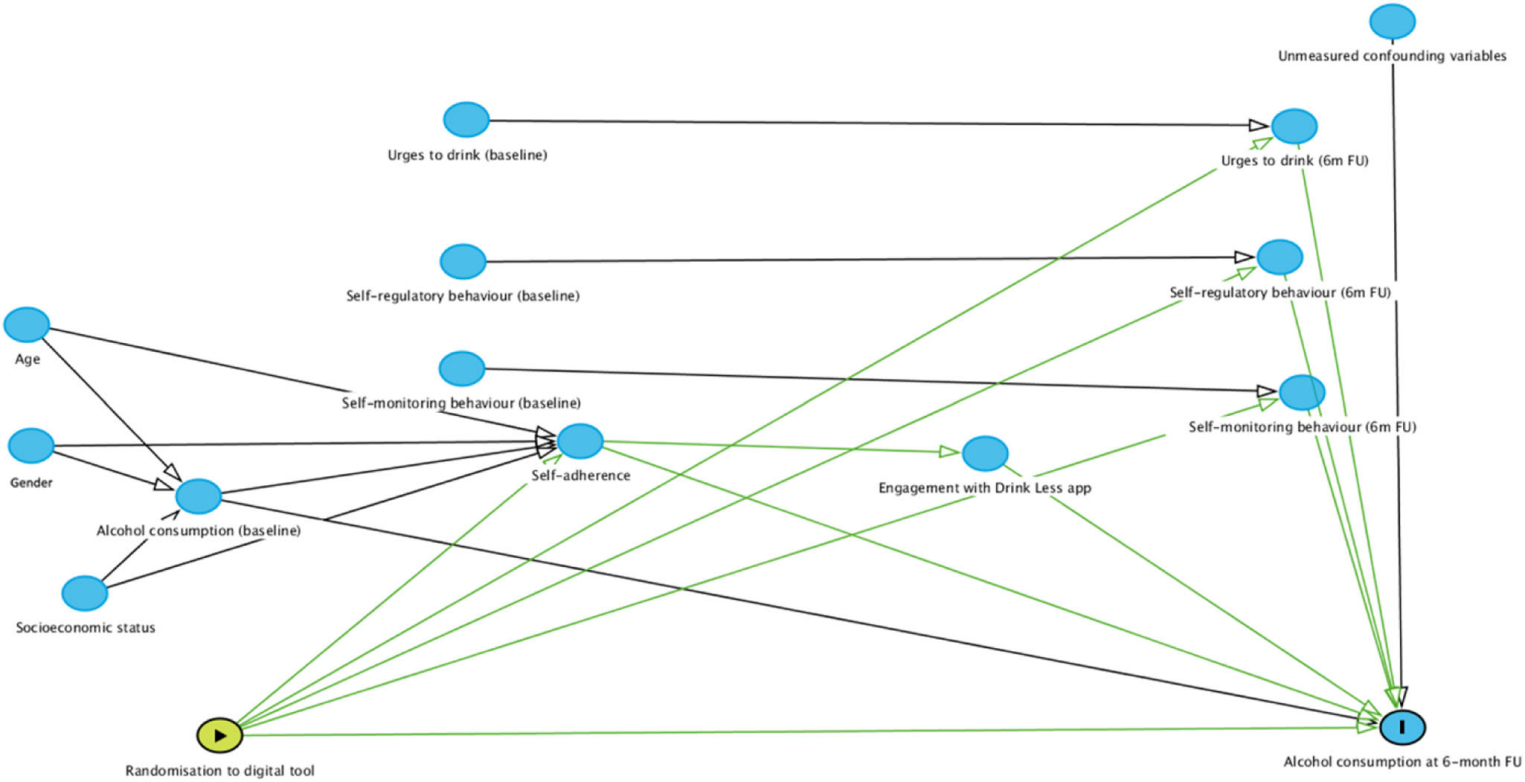
Directed acyclic graph showing potential causal mechanism for the effect of Drink Less on self-reported adherence, self-regulatory and self-monitoring behaviour and urges to drink. Directed acyclic graph showing potential causal mechanism for the effect of randomisation to the Drink Less app on the outcome, weekly alcohol consumption at 6-month follow-up. A series of model-based causal mediation analyses were run for each potential mediator: (1) self-reported adherence, (2) self-regulatory behaviour, (3) self-monitoring behaviour and (4) urges to drink. The unmarked blue ovals refer to ancestors of outcome, the green oval refers to exposure, and the blue oval with an ‘I’ refers to the outcome.

For RQ4 (urges to drink, self-regulatory and self-monitoring behaviour and self-reported adherence as mediators of intervention effectiveness), a series of model-based causal mediation analyses are run to assess the following potential causal mechanisms for the effect of randomisation to the Drink Less app or NHS alcohol advice webpage on the primary outcome: (1) self-reported adherence to the recommended digital tool; (2) self-regulatory behaviour at 6-month follow-up, adjusting for baseline; (3) self-monitoring behaviour at 6-month follow-up, adjusting for baseline; (4) urges to drink at 6-month follow-up, adjusting for baseline. Two statistical models are specified, where we test the causal relationship between (1) the treatment status and the mediator (mediator model) and (2) the treatment status and the outcome (outcome model) (see Fig. 3 for the directed acyclic graph; DAG):

1. The mediator model, where the mediator of interest (mediator) is modelled as a function of the recommendation of the digital tool (treatment status) and pre-treatment covariates (baseline alcohol consumption, age, gender, education level and occupation).
2. The outcome model, where the outcome variable is alcohol consumption at 6-month follow-up, and the explanatory variables include the mediator, treatment status, and the same set of pre-treatment covariates as in the mediator model.

The key identifying assumption is sequential ignorability which implies that, conditional on covariates, there is no unmeasured confounding of the treatment-mediator, treatment-outcome and mediator-outcome relationships. This assumption is satisfied if the treatment is randomised, as is the case with this analysis.

The ‘mediation’ package in R^37^ and the *mediate* function are used to estimate the average causal mediation effects (ACME) and the average direct effects (ADE), which represent the population averages of the causal mediation and direct effects. The ACME is the estimated average causal mediation effect in alcohol reduction among the treatment status (i.e., intervention vs. comparator group) as a result of the mediator, rather than ‘directly’ from the treatment. The ADE represents the average change in the outcome variable directly influenced by the treatment status. The mediate function is run on each of the 20 multiple imputed datasets and then combined the components of the output using the *amelidiate* function (this function does not pass the information required for calculation of *p* values).

For RQ5 (extent of behavioural engagement as a mediator of the effect of self-reported adherence on intervention effectiveness in the intervention group), a series of model-based causal mediation analyses are run to assess, among participants in the intervention group, the following measures of engagement as a causal mechanism for the effect of self-reported adherence (whether the participant downloaded Drink Less) on the primary outcome: (1) depth; (2) frequency; (3) duration; and (4) amount of use over the 6-month period from the date of recommendation. Two statistical models are specified:

1. The mediator model, where the mediator of interest (extent of engagement) is modelled as a function of self-reported adherence (treatment status) and pre-treatment covariates (baseline alcohol consumption, age, gender, education level and occupation).
2. The outcome model, where the outcome variable is alcohol consumption at 6-month follow-up, and the explanatory variables include the mediator, treatment status, and the same set of pre-treatment covariates as in the mediator model.

The average causal mediation effects (ACME) and the average direct effects (ADE) were estimated using the same R packages and procedures as specified above for RQ4.

### Reporting summary

Further information on research design is available in the Nature Research Reporting Summary linked to this article.

### Supplementary information

Supplementary Information

Reporting summary

## Data Availability

The anonymised data and data dictionary are available online at OSF (https://osf.io/2j9df/).

## Code availability

The anonymised code is available online at OSF (https://osf.io/2j9df/).
