# Supplementary material for: Do engagement and behavioural mechanisms underpin the effectiveness of the Drink Less app?
Source: NPJ Digit Med. 2024 Jun 29;7:174. doi: 10.1038/s41746-024-01169-7 (PMC11217434; doi:10.1038/s41746-024-01169-7)
Supplement: Supplementary file 1 — Supplementary Information [file 41746_2024_1169_MOESM1_ESM.pdf]

Supplementary File 1

i) What are the trial participants' urges to drink, motivation to drink less and self-regulatory and self-monitoring behaviour at baseline and 6-month follow-up, stratified by group?

Descriptive statistics for the behavioural measures assessed as potential mechanisms of action (urges to drink; motivation to drink less; self-regulatory and self-monitoring behaviour) at both baseline and 6-month follow-up were reported for all participants, and stratified by group (intervention versus comparator). We ran sensitivity analyses where only those participants responding to 6-month follow-up were included in the analytic sample. Supplementary Table 1 shows descriptive statistics of trial participants' potential mechanisms of action at both baseline and 6-month follow-up, overall and stratified by group, with both multiple imputation and complete cases for the missing data at 6-month follow-up data.

ii) Among participants in the intervention group, how do self-reported adherence, engagement and urges to drink, motivation to drink less, self-regulatory and self-monitoring behaviour differ by sociodemographic and drinking characteristics?

To explore the variation between participants randomised to the intervention group in self-reported adherence, engagement and behavioural characteristics (urges to drink; motivation to drink less; self-regulatory and self-monitoring behaviour) at 6-month follow-up, we reported descriptive statistics for these measures stratified by sociodemographic characteristics and baseline AUDIT scores (grouped into 8-15, 16-19 and 20-40) and COVID-19 measures. We ran sensitivity analyses where only those participants responding to 6-month follow-up were included in the analytic sample.

Self-reported adherence among participants in the intervention group stratified by sociodemographic and drinking characteristics is reported in Supplementary Table 2 (both with multiple imputation and complete cases). Among all participants in the intervention group, their engagement with the Drink Less app is reported in Supplementary Table 3 stratified by sociodemographic and drinking characteristics. Among participants in the intervention group, urges to drink, motivation to drink less, self-regulatory and self-monitoring behaviour are reported in Supplementary Table 4 (using multiple imputation) and Supplementary Table 5 (using complete cases).

iii) Among participants in the comparator group, how do self-reported adherence and urges to drink, motivation to drink less, self-regulatory and self-monitoring behaviour differ by sociodemographic and drinking characteristics?

To explore the variation between participants randomised to the comparator group in self-reported adherence and behavioural characteristics (urges to drink; motivation to drink less; self-regulatory and self-monitoring behaviour) at 6-month follow-up, we reported descriptive statistics for these measures stratified by sociodemographic characteristics and baseline AUDIT scores (grouped into 8-15, 16-19 and 20-40) and COVID-19 measures. We ran sensitivity analyses where only those participants responding to 6-month follow-up were included in the analytic sample.

Self-reported adherence among participants in the comparator group stratified by sociodemographic and drinking characteristics is reported in Supplementary Table 2 (both with multiple imputation and complete cases). Among participants in the comparator group, urges to drink, motivation to drink less, self-regulatory and self-monitoring behaviour are reported in Supplementary Table 6 (using multiple imputation) and Supplementary Table 7 (using complete cases).

Supplementary Table 1: Descriptive statistics for potential mechanisms of action at baseline and 6-month follow-up, overall and stratified by group

Mechanisms of action	**Overall** (n=5,602)	**Comparator group** (n=2,814)	**Intervention group** (n=2,788)
Urges to drink (baseline)			
Not at all	510 (9.1%)	278 (9.9%)	232 (8.3%)
Slight	863 (15%)	458 (16%)	405 (15%)
Moderate	1,543 (28%)	757 (27%)	786 (28%)
Strong	1,469 (26%)	718 (26%)	751 (27%)
Very strong	737 (13%)	363 (13%)	374 (13%)
Extremely strong	480 (8.6%)	240 (8.5%)	240 (8.6%)
Urges to drink (6-month follow-up, multiple imputation)			
Not at all	1310 (23.4%)	641 (22.8%)	669 (24.0%)
Slight	2261 (40.4%)	1157 (41.1%)	1104 (39.6%)
Moderate	1296 (23.1%)	637 (22.6%)	659 (23.6%)
Strong	468 (8.4%)	232 (8.2%)	236 (8.5%)
Very strong	161 (2.9%)	86 (3.1%)	75 (2.7%)
Extremely strong	106 (1.9%)	61 (2.2%)	45 (1.6%)
Urges to drink (6-month follow-up, complete cases)			
Not at all	1,278 (29%)	623 (28%)	655 (31%)
Slight	1,482 (34%)	774 (35%)	708 (33%)
Moderate	852 (20%)	423 (19%)	429 (20%)
Strong	459 (11%)	229 (10%)	230 (11%)
Very strong	161 (3.7%)	86 (3.9%)	75 (3.5%)
Extremely strong	106 (2.4%)	61 (2.8%)	45 (2.1%)
Unknown	1,264	618	646
Motivation to drink less (baseline)			
Really want to and intend to in next month	2,769 (49%)	1,381 (49%)	1,388 (50%)
Really want to and intend to in next 3 months	634 (11%)	327 (12%)	307 (11%)
Want to and hope to soon	971 (17%)	493 (18%)	478 (17%)
Really want to but don't know when I will	585 (10%)	281 (10.0%)	304 (11%)
Want to but haven't thought about when	243 (4.3%)	123 (4.4%)	120 (4.3%)
Think I should but don't really want to	385 (6.9%)	200 (7.1%)	185 (6.6%)
I don't want to	15 (0.3%)	9 (0.3%)	6 (0.2%)
Motivation to drink less (6-month follow-up, multiple imputation)			
Really want to and intend to in next month	1918 (34.2%)	965 (34.3%)	953 (34.2%)
Really want to and intend to in next 3 months	522 (9.3%)	259 (9.2%)	263 (9.4%)
Want to and hope to soon	1481 (26.4%)	744 (26.4%)	737 (26.4%)
Really want to but don't know when I will	712 (12.7%)	356 (12.7%)	356 (12.8%)
Want to but haven't thought about when	184 (3.3%)	97 (3.4%)	87 (3.1%)
Think I should but don't really want to	391 (7.0%)	200 (7.1%)	191 (6.9%)
I don't want to	394 (7.0%)	193 (6.9%)	201 (7.2%)
Motivation to drink less (6-month follow-up, complete cases)			
Really want to and intend to in next month	1,918 (44%)	965 (44%)	953 (45%)
Really want to and intend to in next 3 months	460 (11%)	232 (11%)	228 (11%)
Want to and hope to soon	673 (16%)	344 (16%)	329 (15%)
Really want to but don't know when I will	331 (7.6%)	173 (7.9%)	158 (7.4%)
Want to but haven't thought about when	170 (3.9%)	89 (4.1%)	81 (3.8%)
Think I should but don't really want to	391 (9.0%)	200 (9.1%)	191 (8.9%)
I don't want to	394 (9.1%)	193 (8.8%)	201 (9.4%)
Unknown	1,265	618	647
Self-regulatory behaviour (baseline)			
Not at all	177 (3.2%)	93 (3.3%)	84 (3.0%)
Slightly	1,077 (19%)	557 (20%)	520 (19%)
Moderately	1,940 (35%)	963 (34%)	977 (35%)
Very	1,806 (32%)	896 (32%)	910 (33%)
Extremely	602 (11%)	305 (11%)	297 (11%)
Self-regulatory behaviour (6-month follow-up, multiple imputation)			
Not at all	762 (13.6%)	398 (14.1%)	364 (13.1%)
Slightly	2412 (43.1%)	1227 (43.6%)	1185 (42.5%)
Moderately	1605 (28.7%)	768 (27.3%)	837 (30.0%)
Very	637 (11.4%)	320 (11.4%)	317 (11.4%)
Extremely	186 (3.3%)	101 (3.6%)	85 (3.0%)
Self-regulatory behaviour (6-month follow-up, complete cases)			
Not at all	732 (17%)	378 (17%)	354 (17%)
Slightly	1,657 (38%)	861 (39%)	796 (37%)
Moderately	1,127 (26%)	536 (24%)	591 (28%)
Very	635 (15%)	320 (15%)	315 (15%)
Extremely	186 (4.3%)	101 (4.6%)	85 (4.0%)
Unknown	1,265	618	647
Self-monitoring behaviour (baseline)			
Never	2,758 (49%)	1,401 (50%)	1,357 (49%)
Rarely	1,446 (26%)	727 (26%)	719 (26%)
Sometimes	844 (15%)	400 (14%)	444 (16%)
Very often	366 (6.5%)	185 (6.6%)	181 (6.5%)

Mechanisms of action	**Overall** (n=5,602)	**Comparator group** (n=2,814)	**Intervention group** (n=2,788)
Always	188 (3.4%)	101 (3.6%)	87 (3.1%)
Self-monitoring behaviour (6-month follow-up, multiple imputation)			
Never	1511 (27.0%)	874 (31.1%)	637 (22.8%)
Rarely	2138 (38.2%)	1101 (39.1%)	1037 (37.2%)
Sometimes	966 (17.2%)	433 (15.4%)	533 (19.1%)
Very often	540 (9.6%)	218 (7.7%)	322 (11.5%)
Always	447 (8.0%)	188 (6.7%)	259 (9.3%)
Self-monitoring behaviour (6-month follow-up, complete cases)			
Never	1,324 (31%)	776 (35%)	548 (26%)
Rarely	1,145 (26%)	621 (28%)	524 (24%)
Sometimes	881 (20%)	393 (18%)	488 (23%)
Very often	540 (12%)	218 (9.9%)	322 (15%)
Always	447 (10%)	188 (8.6%)	259 (12%)
Unknown	1,265	618	647

Supplementary Table 2: Self-reported adherence by intervention group stratified by sociodemographic and drinking characteristics

	Intervention group				Comparator group			
	Self-reported adherence, complete cases (n=2,262)		Self-reported adherence, multiple imputation (n=2,788)		Self-reported adherence, complete cases (n=2,345)		Self-reported adherence, multiple imputation	
	% No (n)	% Yes (n)	% No (n)	% Yes (n)	% No (n)	% Yes (n)	% No (n)	% Yes (n)
Age								
18-24	32% (58)	68% (125)	42.1% (91)	57.9% (125)	38% (79)	62% (131)	47% (116)	53% (131)
25-34	23% (137)	77% (471)	34.9% (253)	65.1% (471)	33% (191)	67% (396)	42.5% (294)	57.5% (397)
35-44	17% (100)	83% (475)	34.8% (254)	65.2% (476)	28% (164)	72% (432)	41.7% (310)	58.3% (433)
45-54	18% (88)	82% (402)	35.8% (225)	64.2% (404)	27% (135)	73% (357)	41.2% (251)	58.8% (358)
55-64	22% (66)	78% (239)	34.9% (129)	65.1% (241)	23% (81)	77% (267)	33.2% (133)	66.8% (267)
65+	31% (31)	69% (70)	41.2% (49)	58.8% (70)	29% (33)	71% (79)	36.3% (45)	63.7% (79)
Gender								
Female	21% (270)	79% (1,044)	35.5% (577)	64.5% (1049)	28% (358)	72% (931)	41% (648)	59% (933)
Male	22% (209)	78% (729)	36.6% (420)	63.4% (729)	31% (319)	69% (723)	40.5% (492)	59.5% (724)
Other/ prefer not to say	11% (1)	89% (8)	33.3% (4)	66.7% (8)	55% (6)	45% (5)	64.3% (9)	35.7% (5)
Ethnicity								
White	21% (449)	79% (1,678)	35.9% (944)	64.1% (1683)	29% (636)	71% (1,584)	40.6% (1083)	59.4% (1586)
Ethnic minority	23% (29)	77% (97)	35.8% (54)	64.2% (97)	36% (42)	64% (74)	44.4% (60)	55.6% (75)
Income								
£26,000 or more	20% (350)	80% (1,380)	34.5% (728)	65.5% (1381)	29% (511)	71% (1,245)	40% (831)	60% (1248)
Less than £26,000	24% (130)	76% (402)	40.2% (273)	59.8% (406)	29% (172)	71% (417)	43.3% (318)	56.7% (417)
Social grade								
ABC1	20% (332)	80% (1,368)	33.6% (696)	66.4% (1373)	28% (495)	72% (1,252)	39.8% (828)	60.2% (1254)
C2DE	26% (148)	74% (414)	42.4% (305)	57.6% (414)	31% (188)	69% (410)	43.9% (321)	56.1% (411)
Educational qualification								
Pre-16	29% (23)	71% (57)	43.6% (44)	56.4% (57)	27% (27)	73% (73)	39.2% (47)	60.8% (73)
Post-16	21% (457)	79% (1,725)	35.6% (957)	64.4% (1730)	29% (656)	71% (1,589)	40.9% (1102)	59.1% (1592)
AUDIT category								
8-15	20% (100)	80% (403)	29.3% (167)	70.7% (403)	28% (135)	72% (350)	34.3% (183)	65.7% (350)
16-19	19% (78)	81% (329)	31.6% (152)	68.4% (329)	28% (134)	72% (342)	38.3% (212)	61.7% (342)
20-40	22% (302)	78% (1,050)	39.3% (682)	60.7% (1055)	30% (414)	70% (970)	43.7% (754)	56.3% (973)
Drinking affected by COVID-19								
Yes	22% (232)	78% (821)	22% (232)	78% (821)	28% (300)	72% (770)	28% (300)	72% (770)
No	20% (217)	80% (871)	19.9% (217)	80.1% (871)	29% (328)	71% (798)	29.1% (328)	70.9% (798)
NA	26% (31)	74% (90)	85.3% (552)	14.7% (95)	37% (55)	63% (94)	84.3% (521)	15.7% (97)

Supplementary Table 3: Descriptive statistics for engagement measures among intervention group (n=2,788) over a 6-month period stratified by sociodemographic and drinking characteristics

	**Number of sessions (frequency), (n=2,788)**	**Time on app in minutes (amount), (n=2,788)**	**Number of days used (duration), (n=2,788)**	**Number of unique screens viewed (depth), (n=2,788)**
	Mean (SD)	Mean (SD)	Mean (SD)	Mean (SD)
Age				
18-24	14.1 (37.91)	20.4 (60.04)	11.0 (27.81)	11.0 (12.21)
25-34	23.8 (53.92)	34.3 (92.96)	17.5 (35.86)	14.5 (13.64)
35-44	34.8 (68.59)	52.4 (114.57)	24.7 (44.13)	18.4 (14.41)
45-54	40.9 (67.33)	61.5 (109.52)	30.5 (46.83)	19.2 (15.11)
55-64	51.9 (77.18)	93.6 (155.85)	38.9 (55.03)	21.2 (16.68)
65+	41.3 (71.02)	82.6 (151.04)	32.9 (51.53)	18.3 (17.30)
Gender				
Female	35.6 (63.94)	58.1 (110.47)	26.3 (43.77)	19.1 (15.19)
Male	31.8 (64.71)	47.6 (120.01)	23.7 (44.96)	15.0 (14.22)
Other or prefer not to say	90.8 (164.5)	120.5 (218.02)	48.2 (73.38)	17.5 (18.84)
Ethnicity				
White	34.7 (65.42)	54.7 (116.27)	25.7 (44.67)	17.6 (14.93)
Ethnic minority	27.9 (60.13)	44.8 (99.38)	20.5 (41.3)	13.5 (14.66)
Income				
£26,000 or more	35.6 (65.12)	54.9 (112.51)	26.3 (44.6)	18.0 (14.74)
Less than £26,000	30.1 (64.75)	51.4 (123.43)	22.3 (43.84)	15.4 (15.4)
Social grade				
ABC1	35.6 (65.99)	54.5 (114.59)	26.1 (44.52)	18.0 (14.62)
C2DE	30.2 (62.16)	52.6 (117.21)	23.1 (44.17)	15.7 (15.74)
Educational qualifications				
Pre-16	18.0 (40.73)	32.3 (59.88)	13.6 (28.58)	15.4 (14.37)
Post-16	34.9 (65.72)	54.8 (116.75)	25.8 (44.87)	17.5 (14.96)
AUDIT category				
8-15	38.3 (67.88)	54.0 (114.66)	28.5 (47.2)	16.6 (14.67)
16-19	41.2 (75.04)	60.6 (128.84)	30.3 (49.39)	17.9 (15.22)
20-40	31.0 (60.81)	52.2 (111.4)	22.9 (41.83)	17.5 (14.96)
Drinking affected by COVID-19				
Yes	39.7 (73.39)	63.4 (140.84)	28.2 (46.92)	18.1 (15.26)
No	42.3 (68.94)	64.3 (112.47)	32.2 (49.74)	18.8 (15.39)
NA	11.9 (28.92)	21.5 (48.38)	8.9 (20.25)	13.8 (12.98)

Supplementary Table 4: Descriptive statistics for potential mechanisms of action among intervention group (n=2,788) at 6-month follow-up (using multiple imputation) stratified by sociodemographic and drinking characteristics

	**Urges to drink (n=2,788)**						**Motivation to drink less (n=2,788)**							**Self-regulatory behaviour (n=2,788)**					**Self-monitoring behaviour (n=2,788)**				
	Not at all	Slight	Moderate	Strong	Very strong	Extremely strong	Really want to and intend to in next month	Really want to and intend to in next 3 months	Want to and hope to soon	Really want to but don't know when I will	Want to but haven't thought about when	Think I should but don't really want to	I don't want to	Not at all	Slightly	Moderately	Very	Extremely	Never	Rarely	Sometimes	Very often	Always
Age																							
18-24	38.9% (84)	29.6% (64)	17.1% (37)	8.8% (19)	3.7% (8)	1.9% (4)	20.8% (45)	7.9% (17)	25.9% (56)	11.1% (24)	7.4% (16)	12% (26)	14.8% (32)	27.8% (60)	38% (82)	19.4% (42)	10.6% (23)	4.2% (9)	36.1% (78)	38% (82)	15.3% (33)	7.9% (17)	2.8% (6)
25-34	29.8% (216)	39.5% (286)	20.6% (149)	5% (36)	3.2% (23)	1.9% (14)	29.6% (214)	9.7% (70)	26.2% (190)	13.5% (98)	5.2% (38)	6.4% (46)	9.4% (68)	16.7% (121)	45.4% (329)	26.2% (190)	7.9% (57)	3.7% (27)	31.8% (230)	38.3% (277)	17% (123)	6.2% (45)	6.8% (49)
35-44	20.8% (152)	41.1% (300)	24.9% (182)	9.2% (67)	2.5% (18)	1.5% (11)	35.3% (258)	10.3% (75)	29% (212)	11.9% (87)	2.2% (16)	5.3% (39)	5.9% (43)	9% (66)	44.4% (324)	34.5% (252)	9.9% (72)	2.2% (16)	20.4% (149)	41.1% (300)	17.8% (130)	11.5% (84)	9.2% (67)
45-54	19.2% (121)	43.1% (271)	25.1% (158)	8.9% (56)	2.1% (13)	1.6% (10)	39% (245)	9.5% (60)	26.9% (169)	12.2% (77)	0.8% (5)	6.7% (42)	4.9% (31)	10.7% (67)	42% (264)	29.9% (188)	13.8% (87)	3.7% (23)	17.3% (109)	35.6% (224)	23.5% (148)	14.3% (90)	9.2% (58)
55-64	18.9% (70)	36.5% (135)	28.9% (107)	11.1% (41)	3.2% (12)	1.4% (5)	41.4% (153)	8.9% (33)	23.2% (86)	13.2% (49)	2.4% (9)	7.3% (27)	3.5% (13)	8.9% (33)	37.6% (139)	34.6% (128)	16.5% (61)	2.4% (9)	14.3% (53)	31.9% (118)	18.6% (69)	18.6% (69)	16.5% (61)
65+	21.8% (26)	40.3% (48)	21.8% (26)	14.3% (17)	0.8% (1)	0.8% (1)	31.9% (38)	6.7% (8)	20.2% (24)	17.6% (21)	2.5% (3)	9.2% (11)	11.8% (14)	14.3% (17)	39.5% (47)	31.1% (37)	14.3% (17)	0.8% (1)	15.1% (18)	30.3% (36)	25.2% (30)	14.3% (17)	15.1% (18)
Gender																							
Female	25.8% (419)	41.5% (675)	22.3% (362)	6.8% (111)	2.3% (37)	1.4% (22)	35.7% (580)	9.3% (151)	25.6% (417)	11.9% (193)	2.8% (45)	6.8% (111)	7.9% (129)	14% (227)	44.5% (723)	28.4% (462)	10.6% (172)	2.6% (42)	22.1% (359)	36% (586)	20.3% (330)	12.6% (205)	9% (146)
Male	21.6% (248)	36.9% (424)	25.5% (293)	10.7% (123)	3.3% (38)	2% (23)	32.2% (370)	9.6% (110)	27.6% (317)	14.1% (162)	3.6% (41)	6.9% (79)	6.1% (70)	11.7% (134)	39.9% (458)	32.3% (371)	12.4% (143)	3.7% (43)	23.8% (274)	38.9% (447)	17.5% (201)	10.2% (117)	9.6% (110)
Other or prefer not to say	16.7% (2)	33.3% (4)	33.3% (4)	16.7% (2)	0% (0)	0% (0)	16.7% (2)	16.7% (2)	25% (3)	8.3% (1)	8.3% (1)	8.3% (1)	16.7% (2)	25% (3)	25% (3)	33.3% (4)	16.7% (2)	0% (0)	33.3% (4)	33.3% (4)	8.3% (1)	0% (0)	25% (3)
Ethnicity																							
White	23.8% (625)	39.9% (1049)	23.6% (619)	8.4% (220)	2.7% (71)	1.6% (43)	34.7% (911)	9.3% (243)	26.3% (692)	13% (342)	3% (79)	6.7% (177)	7% (183)	12.7% (333)	42.7% (1121)	29.9% (786)	11.7% (307)	3% (80)	23% (603)	37.3% (979)	18.8% (494)	11.7% (307)	9.3% (244)
Ethnic minority	27.2% (41)	33.8% (51)	24.5% (37)	10.6% (16)	2.6% (4)	1.3% (2)	25.8% (39)	12.6% (19)	27.2% (41)	9.3% (14)	4.6% (7)	9.3% (14)	11.3% (17)	20.5% (31)	38.4% (58)	31.8% (48)	6% (9)	3.3% (5)	19.9% (30)	36.4% (55)	23.8% (36)	9.9% (15)	9.9% (15)
Income																							
£26,000 or more	24.6% (519)	40.4% (853)	23.2% (490)	8.1% (171)	2.4% (50)	1.2% (26)	34.6% (730)	9.7% (204)	25.7% (542)	12.5% (263)	3% (63)	7.3% (155)	7.2% (152)	12.9% (272)	43.4% (915)	30% (632)	11% (233)	2.7% (57)	21.7% (458)	37% (780)	19.7% (415)	11.8% (249)	9.8% (207)
Less than £26,000	22.1% (150)	37% (251)	24.9% (169)	9.6% (65)	3.7% (25)	2.8% (19)	32.8% (223)	8.7% (59)	28.7% (195)	13.7% (93)	3.5% (24)	5.3% (36)	7.2% (49)	13.5% (92)	39.8% (270)	30.2% (205)	12.4% (84)	4.1% (28)	26.4% (179)	37.8% (257)	17.4% (118)	10.8% (73)	7.7% (52)
Social grade																							
ABC1	24.6% (510)	40.6% (840)	22.7% (469)	8.3% (172)	2.7% (55)	1.1% (23)	34.5% (714)	9.9% (204)	27.2% (563)	11.6% (240)	2.9% (60)	6.9% (143)	7% (145)	12.9% (266)	43.3% (896)	30.1% (622)	11.1% (229)	2.7% (56)	21.4% (442)	37.5% (776)	19.7% (407)	11.8% (244)	9.7% (200)
C2DE	22.1% (159)	36.7% (264)	26.4% (190)	8.9% (64)	2.8% (20)	3.1% (22)	33.2% (239)	8.2% (59)	24.2% (174)	16.1% (116)	3.8% (27)	6.7% (48)	7.8% (56)	13.6% (98)	40.2% (289)	29.9% (215)	12.2% (88)	4% (29)	27.1% (195)	36.3% (261)	17.5% (126)	10.8% (78)	8.2% (59)
Educational qualifications																							
Pre-16	28.7% (29)	33.7% (34)	24.8% (25)	7.9% (8)	3% (3)	2% (2)	33.7% (34)	5% (5)	25.7% (26)	11.9% (12)	8.9% (9)	8.9% (9)	5.9% (6)	18.8% (19)	44.6% (45)	20.8% (21)	11.9% (12)	4% (4)	37.6% (38)	35.6% (36)	19.8% (20)	5% (5)	2% (2)
Post-16	23.8% (640)	39.8% (1070)	23.6% (634)	8.5% (228)	2.7% (72)	1.6% (43)	34.2% (919)	9.6% (258)	26.5% (711)	12.8% (344)	2.9% (78)	6.8% (182)	7.3% (195)	12.8% (345)	42.4% (1140)	30.4% (816)	11.4% (305)	3% (81)	22.3% (599)	37.3% (1001)	19.1% (513)	11.8% (317)	9.6% (257)
AUDIT category																							
8-15	36.1% (206)	44.7% (255)	14.6% (83)	4% (23)	0.5% (3)	0% (0)	26.7% (152)	6.8% (39)	22.1% (126)	8.1% (46)	4.9% (28)	10.4% (59)	21.1% (120)	31.6% (180)	51.9% (296)	14.2% (81)	1.8% (10)	0.5% (3)	20.5% (117)	34.9% (199)	20.7% (118)	12.5% (71)	11.4% (65)
16-19	28.7% (138)	42.4% (204)	20% (96)	7.5% (36)	0.8% (4)	0.6% (3)	33.3% (160)	11.6% (56)	25.4% (122)	11% (53)	3.3% (16)	9.8% (47)	5.6% (27)	15% (72)	54.3% (261)	23.3% (112)	6.9% (33)	0.6% (3)	21.8% (105)	32.6% (157)	21% (101)	12.1% (58)	12.5% (60)
20-40	18.7% (325)	37.1% (645)	27.6% (480)	10.2% (177)	3.9% (68)	2.4% (42)	36.9% (641)	9.7% (168)	28.2% (489)	14.8% (257)	2.5% (43)	4.9% (85)	3.1% (54)	6.4% (112)	36.2% (628)	37.1% (644)	15.8% (274)	4.5% (79)	23.9% (415)	39.2% (681)	18.1% (314)	11.1% (193)	7.7% (134)
Drinking affected by COVID-19																							
Yes	26.1% (275)	34.9% (367)	23% (242)	10.3% (108)	3.4% (36)	2.4% (25)	46.8% (493)	11.5% (121)	16.2% (171)	6.6% (70)	3.7% (39)	9.2% (97)	5.9% (62)	12% (126)	40.4% (425)	29.7% (313)	14.2% (150)	3.7% (39)	24.2% (255)	25.3% (266)	25.5% (269)	14.4% (152)	10.5% (111)
No	34.8% (379)	31.3% (341)	17.2% (187)	11.2% (122)	3.6% (39)	1.8% (20)	42.3% (460)	9.8% (107)	14.5% (158)	8.1% (88)	3.9% (42)	8.6% (94)	12.8% (139)	21% (228)	34.1% (371)	25.6% (278)	15.2% (165)	4.2% (46)	26.9% (293)	23.7% (258)	20.1% (219)	15.6% (170)	13.6% (148)
NA	2.3% (15)	61.2% (396)	35.5% (230)	0.9% (6)	0% (0)	0% (0)	0% (0)	5.4% (35)	63.1% (408)	30.6% (198)	0.9% (6)	0% (0)	0% (0)	1.5% (10)	60.1% (389)	38% (246)	0.3% (2)	0% (0)	13.8% (89)	79.3% (513)	7% (45)	0% (0)	0% (0)

Supplementary Table 5: Descriptive statistics for potential mechanisms of action among intervention group at 6-month follow-up (using complete cases) stratified by sociodemographic and drinking characteristics

	Urges to drink (n=2,142)						Motivation to drink less (n=2,141)							Self-regulatory behaviour (n=2,141)					Self-monitoring behaviour (n=2,141)				
	Not at all	Slight	Moderate	Strong	Very strong	Extremely strong	Really want to and intend to in next month	Really want to and intend to in next 3 months	Want to and hope to soon	Really want to but don't know when I will	Want to but haven't thought about when	Think I should but don't really want to	I don't want to	Not at all	Slightly	Moderately	Very	Extremely	Never	Rarely	Sometimes	Very often	Always
Age																							
18-24	45% (80)	25% (44)	12% (22)	11% (19)	4.5% (8)	2.3% (4)	25% (45)	9.0% (16)	22% (39)	2.8% (5)	7.9% (14)	15% (26)	18% (32)	33% (59)	33% (58)	16% (29)	12% (22)	5.1% (9)	38% (68)	31% (54)	18% (32)	9.6% (17)	3.4% (6)
25-34	37% (211)	33% (186)	18% (103)	6.1% (35)	4.0% (23)	2.4% (14)	37% (214)	12% (67)	18% (101)	7.2% (41)	6.1% (35)	8.0% (46)	12% (68)	21% (118)	40% (230)	25% (141)	9.8% (56)	4.7% (27)	35% (200)	28% (159)	21% (119)	7.9% (45)	8.6% (49)
35-44	28% (150)	33% (182)	22% (120)	12% (63)	3.3% (18)	2.0% (11)	47% (258)	12% (65)	16% (88)	6.4% (35)	2.9% (16)	7.2% (39)	7.9% (43)	12% (63)	41% (222)	31% (171)	13% (72)	2.9% (16)	22% (120)	28% (151)	22% (122)	15% (84)	12% (67)
45-54	25% (118)	37% (169)	21% (98)	12% (55)	2.8% (13)	2.2% (10)	53% (245)	9.5% (44)	13% (61)	7.4% (34)	1.1% (5)	9.1% (42)	6.7% (31)	14% (65)	35% (164)	27% (123)	19% (87)	5.0% (23)	21% (95)	19% (87)	29% (132)	19% (90)	13% (58)
55-64	24% (70)	31% (90)	25% (72)	14% (41)	4.1% (12)	1.7% (5)	53% (153)	9.7% (28)	11% (31)	10% (30)	2.8% (8)	9.3% (27)	4.5% (13)	11% (32)	31% (89)	34% (99)	21% (61)	3.1% (9)	16% (47)	19% (54)	20% (59)	24% (69)	21% (61)
65+	27% (26)	39% (37)	15% (14)	18% (17)	1.0% (1)	1.0% (1)	40% (38)	8.3% (8)	9.4% (9)	14% (13)	3.1% (3)	11% (11)	15% (14)	18% (17)	34% (33)	29% (28)	18% (17)	1.0% (1)	19% (18)	20% (19)	25% (24)	18% (17)	19% (18)
Gender																							
Female	33% (411)	34% (429)	20% (246)	8.6% (108)	3.0% (37)	1.8% (22)	46% (580)	10% (128)	15% (192)	5.7% (71)	3.4% (42)	8.9% (111)	10% (129)	18% (220)	39% (485)	27% (334)	14% (172)	3.4% (42)	25% (311)	23% (288)	24% (303)	16% (205)	12% (146)
Male	28% (242)	31% (276)	21% (181)	14% (120)	4.3% (38)	2.6% (23)	42% (370)	11% (98)	15% (136)	9.9% (87)	4.4% (39)	9.0% (79)	8.0% (70)	15% (131)	35% (309)	29% (254)	16% (142)	4.9% (43)	27% (233)	27% (235)	21% (184)	13% (117)	13% (110)
Other or prefer not to say	25% (2)	25% (2)	25% (2)	25% (2)	0% (0)	0% (0)	25% (2)	25% (2)	13% (1)	0% (0)	0% (0)	13% (1)	25% (2)	38% (3)	13% (1)	38% (3)	13% (1)	0% (0)	50% (4)	13% (1)	0% (0)	0% (0)	38% (3)
Ethnicity																							
White	30% (612)	33% (673)	20% (401)	11% (214)	3.5% (71)	2.1% (43)	45% (911)	10% (208)	15% (308)	7.5% (151)	3.7% (75)	8.8% (177)	9.1% (183)	16% (323)	37% (754)	27% (551)	15% (305)	4.0% (80)	26% (518)	25% (494)	22% (450)	15% (307)	12% (244)
Ethnic minority	33% (40)	27% (33)	21% (26)	13% (16)	3.3% (4)	1.7% (2)	32% (39)	16% (19)	16% (19)	5.8% (7)	5.0% (6)	12% (14)	14% (17)	26% (31)	31% (38)	31% (38)	7.4% (9)	4.1% (5)	22% (27)	24% (29)	29% (35)	12% (15)	12% (15)
Income																							
£26,000 or more	31% (509)	34% (556)	20% (331)	10% (166)	3.1% (50)	1.6% (26)	45% (730)	11% (176)	15% (240)	7.6% (125)	3.6% (59)	9.5% (155)	9.3% (152)	16% (265)	39% (631)	28% (452)	14% (232)	3.5% (57)	24% (401)	25% (403)	23% (377)	15% (249)	13% (207)
Less than £26,000	29% (146)	30% (152)	19% (98)	13% (64)	5.0% (25)	3.8% (19)	44% (223)	10% (52)	18% (89)	6.5% (33)	4.4% (22)	7.1% (36)	9.7% (49)	18% (89)	33% (165)	28% (139)	16% (83)	5.6% (28)	29% (147)	24% (121)	22% (111)	14% (73)	10% (52)
Social grade																							
ABC1	31% (502)	34% (541)	20% (325)	10% (167)	3.4% (55)	1.4% (23)	44% (714)	11% (179)	16% (262)	7.0% (113)	3.5% (56)	8.9% (143)	9.0% (145)	16% (259)	38% (616)	28% (452)	14% (229)	3.5% (56)	24% (388)	25% (409)	23% (371)	15% (244)	12% (200)
C2DE	29% (153)	32% (167)	20% (104)	12% (63)	3.8% (20)	4.2% (22)	45% (239)	9.3% (49)	13% (67)	8.5% (45)	4.7% (25)	9.1% (48)	11% (56)	18% (95)	34% (180)	26% (139)	16% (86)	5.5% (29)	30% (160)	22% (115)	22% (117)	15% (78)	11% (59)
Educational qualifications																							
Pre-16	36% (28)	29% (22)	18% (14)	10% (8)	3.9% (3)	2.6% (2)	44% (34)	3.9% (3)	14% (11)	6.5% (5)	12% (9)	12% (9)	7.8% (6)	25% (19)	42% (32)	14% (11)	14% (11)	5.2% (4)	38% (29)	29% (22)	25% (19)	6.5% (5)	2.6% (2)
Post-16	30% (627)	33% (686)	20% (415)	11% (222)	3.5% (72)	2.1% (43)	45% (919)	11% (225)	15% (318)	7.4% (153)	3.5% (72)	8.8% (182)	9.4% (195)	16% (335)	37% (764)	28% (580)	15% (304)	3.9% (81)	25% (519)	24% (502)	23% (469)	15% (317)	12% (257)
AUDIT category																							
8-15	42% (205)	39% (189)	14% (69)	4.7% (23)	0.6% (3)	0% (0)	31% (152)	7.8% (38)	15% (75)	3.9% (19)	5.3% (26)	12% (59)	25% (120)	36% (177)	46% (226)	15% (73)	2.0% (10)	0.6% (3)	21% (105)	28% (136)	23% (112)	15% (71)	13% (65)
16-19	34% (133)	36% (142)	18% (72)	9.2% (36)	1.0% (4)	0.8% (3)	41% (160)	13% (50)	17% (68)	5.9% (23)	3.8% (15)	12% (47)	6.9% (27)	18% (70)	49% (192)	24% (92)	8.5% (33)	0.8% (3)	24% (94)	23% (89)	23% (89)	15% (58)	15% (60)
20-40	25% (317)	30% (377)	23% (288)	14% (171)	5.4% (68)	3.3% (42)	51% (641)	11% (140)	15% (186)	9.2% (116)	3.2% (40)	6.7% (85)	4.3% (54)	8.5% (107)	30% (378)	34% (426)	22% (272)	6.3% (79)	28% (349)	24% (299)	23% (287)	15% (193)	11% (134)
Drinking affected by COVID-19																							
Yes	26% (275)	35% (367)	23% (242)	10% (108)	3.4% (36)	2.4% (25)	47% (493)	11% (121)	16% (171)	6.6% (70)	3.7% (39)	9.2% (97)	5.9% (62)	12% (126)	40% (425)	30% (313)	14% (150)	3.7% (39)	24% (255)	25% (266)	26% (269)	14% (152)	11% (111)
No	35% (379)	31% (341)	17% (187)	11% (122)	3.6% (39)	1.8% (20)	42% (460)	9.8% (107)	15% (158)	8.1% (88)	3.9% (42)	8.6% (94)	13% (139)	21% (228)	34% (371)	26% (278)	15% (165)	4.2% (46)	27% (293)	24% (258)	20% (219)	16% (170)	14% (148)
NA	0% (0)	0% (0)	0% (0)	0% (0)	0% (0)	0% (0)	0% (0)	0% (0)	0% (0)	0% (0)	0% (0)	0% (0)	0% (0)	0% (0)	0% (0)	0% (0)	0% (0)	0% (0)	0% (0)	0% (0)	0% (0)	0% (0)	0% (0)

Supplementary Table 6: Descriptive statistics for potential mechanisms of action among comparator group (n=2,814) at 6-month follow-up (using multiple imputation) stratified by sociodemographic and drinking characteristics

	**Urges to drink (n=2,814)**						**Motivation to drink less (n=2,814)**							**Self-regulatory behaviour (n=2,814)**					**Self-monitoring behaviour (n=2,814)**				
	Not at all	Slight	Moderate	Strong	Very strong	Extremely strong	Really want to and intend to in next month	Really want to and intend to in next 3 months	Want to and hope to soon	Really want to but don't know when I will	Want to but haven't thought about when	Think I should but don't really want to	I don't want to	Not at all	Slightly	Moderately	Very	Extremely	Never	Rarely	Sometimes	Very often	Always
Age																							
18-24	28.3% (70)	44.5% (110)	13.8% (34)	6.5% (16)	4.9% (12)	2% (5)	23.9% (59)	8.5% (21)	27.5% (68)	10.9% (27)	6.9% (17)	10.1% (25)	12.1% (30)	21.9% (54)	42.9% (106)	23.1% (57)	8.5% (21)	3.6% (9)	44.5% (110)	37.7% (93)	11.3% (28)	4.5% (11)	2% (5)
25-34	27.2% (188)	43.7% (302)	18.8% (130)	5.8% (40)	2.3% (16)	2.2% (15)	27.5% (190)	9.7% (67)	28.1% (194)	12.2% (84)	5.5% (38)	7.1% (49)	10% (69)	17.1% (118)	51.4% (355)	21.3% (147)	7.8% (54)	2.5% (17)	37.3% (258)	41.8% (289)	12% (83)	4.5% (31)	4.3% (30)
35-44	21.7% (161)	39% (290)	26% (193)	8.1% (60)	2.8% (21)	2.4% (18)	36.6% (272)	8.5% (63)	28% (208)	12.8% (95)	2.8% (21)	5.8% (43)	5.5% (41)	12.1% (90)	41.9% (311)	30.8% (229)	11.3% (84)	3.9% (29)	31.6% (235)	41.6% (309)	13.6% (101)	7.5% (56)	5.7% (42)
45-54	18.6% (113)	41.2% (251)	24.5% (149)	10.2% (62)	3.3% (20)	2.3% (14)	37.8% (230)	8.4% (51)	27.8% (169)	15.1% (92)	2.5% (15)	4.8% (29)	3.8% (23)	9.7% (59)	42.7% (260)	30.2% (184)	13.3% (81)	4.1% (25)	26.3% (160)	40.6% (247)	17.4% (106)	7.6% (46)	8.2% (50)
55-64	20% (80)	38.2% (153)	25.5% (102)	11.5% (46)	3% (12)	1.8% (7)	42% (168)	11.8% (47)	19.8% (79)	10.2% (41)	0.8% (3)	10.5% (42)	5% (20)	14.5% (58)	36.2% (145)	28% (112)	17% (68)	4.2% (17)	21.5% (86)	32% (128)	21.2% (85)	14.2% (57)	11% (44)
65+	23.4% (29)	41.1% (51)	23.4% (29)	6.5% (8)	4% (5)	1.6% (2)	37.1% (46)	8.1% (10)	21% (26)	13.7% (17)	2.4% (3)	9.7% (12)	8.1% (10)	15.3% (19)	40.3% (50)	31.5% (39)	9.7% (12)	3.2% (4)	20.2% (25)	28.2% (35)	24.2% (30)	13.7% (17)	13.7% (17)
Gender																							
Female	23.9% (378)	43.3% (684)	21.3% (336)	7.2% (114)	2.2% (35)	2.2% (34)	35.3% (558)	8.9% (140)	25% (396)	13.6% (215)	3.4% (53)	6.8% (108)	7% (111)	13.7% (217)	45.9% (726)	25.9% (409)	10.9% (172)	3.6% (57)	30.1% (476)	39% (616)	15.3% (242)	8.5% (135)	7.1% (112)
Male	21.6% (263)	38.5% (468)	24.3% (296)	9.4% (114)	3.9% (48)	2.2% (27)	33.2% (404)	9.7% (118)	28.2% (343)	11.2% (136)	3.5% (43)	7.5% (91)	6.7% (81)	14.8% (180)	40.9% (497)	29% (353)	11.8% (143)	3.5% (43)	32.5% (395)	39.1% (475)	15.5% (189)	6.7% (81)	6.2% (76)
Other or prefer not to say	0% (0)	28.6% (4)	28.6% (4)	21.4% (3)	21.4% (3)	0% (0)	21.4% (3)	7.1% (1)	14.3% (2)	35.7% (5)	7.1% (1)	7.1% (1)	7.1% (1)	7.1% (1)	14.3% (2)	35.7% (5)	35.7% (5)	7.1% (1)	21.4% (3)	57.1% (8)	7.1% (1)	14.3% (2)	0% (0)
Ethnicity																							
White	22.7% (606)	40.9% (1092)	22.9% (612)	8.3% (222)	3% (79)	2.2% (58)	34% (908)	9.1% (242)	26.5% (706)	12.9% (343)	3.4% (91)	7.3% (195)	6.9% (184)	13.7% (366)	43.7% (1167)	27.6% (736)	11.4% (303)	3.6% (97)	31% (828)	38.9% (1037)	15.5% (414)	7.9% (211)	6.7% (179)
Ethnic minority	24.4% (33)	44.4% (60)	17% (23)	6.7% (9)	5.2% (7)	2.2% (3)	39.3% (53)	11.9% (16)	25.2% (34)	9.6% (13)	3.7% (5)	3.7% (5)	6.7% (9)	21.5% (29)	41.5% (56)	22.2% (30)	12.6% (17)	2.2% (3)	33.3% (45)	43.7% (59)	12.6% (17)	4.4% (6)	5.9% (8)
Income																							
£26,000 or more	23.1% (481)	42.1% (875)	21.8% (454)	8.5% (176)	2.8% (58)	1.7% (35)	34.4% (716)	9.4% (196)	27.4% (569)	11.8% (245)	3% (62)	7.3% (151)	6.7% (140)	13.9% (289)	44.6% (927)	27.1% (564)	11.1% (231)	3.3% (68)	30.6% (636)	38.3% (796)	16.3% (338)	8.2% (170)	6.7% (139)
Less than £26,000	21.8% (160)	38.4% (282)	24.9% (183)	7.6% (56)	3.8% (28)	3.5% (26)	33.9% (249)	8.6% (63)	23.8% (175)	15.1% (111)	4.8% (35)	6.7% (49)	7.2% (53)	14.8% (109)	40.8% (300)	27.8% (204)	12.1% (89)	4.5% (33)	32.4% (238)	41.5% (305)	12.9% (95)	6.5% (48)	6.7% (49)
Social grade																							
ABC1	23.9% (498)	41.8% (871)	22% (458)	7.7% (160)	2.6% (54)	2% (41)	35.1% (731)	9.3% (193)	26.8% (558)	11.9% (248)	3.3% (69)	6.8% (142)	6.8% (141)	14.1% (293)	45.1% (938)	26.4% (550)	11% (229)	3.5% (72)	29.5% (615)	40.3% (839)	15.9% (330)	8.2% (170)	6.1% (128)
C2DE	19.5% (143)	39.1% (286)	24.5% (179)	9.8% (72)	4.4% (32)	2.7% (20)	32% (234)	9% (66)	25.4% (186)	14.8% (108)	3.8% (28)	7.9% (58)	7.1% (52)	14.3% (105)	39.5% (289)	29.8% (218)	12.4% (91)	4% (29)	35.4% (259)	35.8% (262)	14.1% (103)	6.6% (48)	8.2% (60)
Educational qualifications																							
Pre-16	25.8% (31)	36.7% (44)	20% (24)	9.2% (11)	7.5% (9)	0.8% (1)	31.7% (38)	6.7% (8)	31.7% (38)	10% (12)	8.3% (10)	8.3% (10)	3.3% (4)	13.3% (16)	47.5% (57)	23.3% (28)	11.7% (14)	4.2% (5)	35% (42)	40.8% (49)	10% (12)	8.3% (10)	5.8% (7)
Post-16	22.6% (610)	41.3% (1113)	22.8% (613)	8.2% (221)	2.9% (77)	2.2% (60)	34.4% (927)	9.3% (251)	26.2% (706)	12.8% (344)	3.2% (87)	7.1% (190)	7% (189)	14.2% (382)	43.4% (1170)	27.5% (740)	11.4% (306)	3.6% (96)	30.9% (832)	39% (1052)	15.6% (421)	7.7% (208)	6.7% (181)
AUDIT category																							
8-15	32.5% (173)	51.2% (273)	11.3% (60)	4.3% (23)	0.6% (3)	0.2% (1)	28.1% (150)	6.9% (37)	23.6% (126)	9.4% (50)	6.2% (33)	9.8% (52)	15.9% (85)	33.2% (177)	53.5% (285)	9.6% (51)	2.8% (15)	0.9% (5)	30% (160)	37.9% (202)	16.7% (89)	8.3% (44)	7.1% (38)
16-19	28.9% (160)	45.7% (253)	18.6% (103)	5.4% (30)	1.1% (6)	0.4% (2)	31% (172)	9.4% (52)	26.7% (148)	10.8% (60)	4.7% (26)	8.7% (48)	8.7% (48)	18.8% (104)	52.2% (289)	22.9% (127)	5.4% (30)	0.7% (4)	31.4% (174)	38.1% (211)	15% (83)	8.5% (47)	7% (39)
20-40	17.8% (308)	36.5% (631)	27.4% (474)	10.4% (179)	4.5% (77)	3.4% (58)	37.2% (643)	9.8% (170)	27.2% (470)	14.2% (246)	2.2% (38)	5.8% (100)	3.5% (60)	6.8% (117)	37.8% (653)	34.2% (590)	15.9% (275)	5.3% (92)	31.3% (540)	39.8% (688)	15.1% (261)	7.4% (127)	6.4% (111)
Drinking affected by COVID-19																							
Yes	25.9% (277)	34.3% (367)	20.6% (220)	11.7% (125)	4.5% (48)	3.1% (33)	45.4% (486)	10.9% (117)	16.8% (180)	8% (86)	4.5% (48)	8.5% (91)	5.8% (62)	12.9% (138)	40.8% (437)	27% (289)	15% (160)	4.3% (46)	36.2% (387)	28.2% (302)	18.9% (202)	9.9% (106)	6.8% (73)
No	30.7% (346)	36.1% (407)	18% (203)	9.2% (104)	3.4% (38)	2.5% (28)	42.5% (479)	10.2% (115)	14.6% (164)	7.7% (87)	3.6% (41)	9.7% (109)	11.6% (131)	21.3% (240)	37.7% (424)	21.9% (247)	14.2% (160)	4.9% (55)	34.5% (389)	28.3% (319)	17% (191)	9.9% (112)	10.2% (115)
NA	2.9% (18)	62% (383)	34.6% (214)	0.5% (3)	0% (0)	0% (0)	0% (0)	4.4% (27)	64.7% (400)	29.6% (183)	1.3% (8)	0% (0)	0% (0)	3.2% (20)	59.2% (366)	37.5% (232)	0% (0)	0% (0)	15.9% (98)	77.7% (480)	6.5% (40)	0% (0)	0% (0)

Supplementary Table 7: Descriptive statistics for potential mechanisms of action among comparator group (n=2,196) at 6-month follow-up (using complete case) stratified by sociodemographic and drinking characteristics

	Urges to drink (n=2,196)						Motivation to drink less (n=2,196)							Self-regulatory behaviour (n=2,196)					Self-monitoring behaviour (n=2,196)				
	Not at all	Slight	Moderate	Strong	Very strong	Extremely strong	Really want to and intend to in next month	Really want to and intend to in next 3 months	Want to and hope to soon	Really want to but don't know when I will	Want to but haven't thought about when	Think I should but don't really want to	I don't want to	Not at all	Slightly	Moderately	Very	Extremely	Never	Rarely	Sometimes	Very often	Always
Age																							
18-24	35% (70)	36% (71)	13% (25)	8.0% (16)	6.0% (12)	2.5% (5)	30% (59)	10% (20)	20% (39)	5.5% (11)	7.5% (15)	13% (25)	15% (30)	26% (52)	40% (79)	19% (38)	11% (21)	4.5% (9)	50% (99)	29% (57)	14% (27)	5.5% (11)	2.5% (5)
25-34	34% (183)	38% (204)	16% (86)	7.0% (38)	3.0% (16)	2.8% (15)	35% (190)	12% (64)	19% (104)	5.5% (30)	6.6% (36)	9.0% (49)	13% (69)	20% (111)	49% (263)	18% (97)	10.0% (54)	3.1% (17)	41% (221)	33% (180)	15% (80)	5.7% (31)	5.5% (30)
35-44	27% (153)	33% (186)	22% (122)	11% (59)	3.8% (21)	3.2% (18)	49% (272)	9.3% (52)	16% (89)	7.3% (41)	3.8% (21)	7.7% (43)	7.3% (41)	15% (84)	37% (209)	27% (153)	15% (84)	5.2% (29)	36% (200)	30% (168)	17% (93)	10% (56)	7.5% (42)
45-54	24% (109)	35% (158)	20% (93)	14% (62)	4.4% (20)	3.1% (14)	50% (230)	9.9% (45)	14% (64)	11% (52)	2.9% (13)	6.4% (29)	5.0% (23)	12% (55)	37% (169)	28% (126)	18% (81)	5.5% (25)	33% (149)	26% (118)	20% (93)	10% (46)	11% (50)
55-64	23% (79)	35% (118)	22% (75)	14% (46)	3.6% (12)	2.1% (7)	50% (168)	12% (41)	10% (35)	8.6% (29)	0.6% (2)	12% (42)	5.9% (20)	17% (57)	31% (105)	27% (90)	20% (68)	5.0% (17)	25% (83)	23% (79)	22% (74)	17% (57)	13% (44)
65+	28% (29)	36% (37)	21% (22)	7.8% (8)	4.9% (5)	1.9% (2)	45% (46)	9.7% (10)	13% (13)	9.7% (10)	1.9% (2)	12% (12)	9.7% (10)	18% (19)	35% (36)	31% (32)	12% (12)	3.9% (4)	23% (24)	18% (19)	25% (26)	17% (17)	17% (17)
Gender																							
Female	30% (366)	37% (441)	18% (214)	9.5% (114)	2.9% (35)	2.8% (34)	46% (558)	9.8% (118)	14% (165)	7.8% (94)	4.2% (50)	9.0% (108)	9.2% (111)	17% (204)	42% (500)	23% (271)	14% (172)	4.7% (57)	35% (418)	27% (325)	18% (214)	11% (135)	9.3% (112)
Male	26% (257)	34% (330)	21% (206)	11% (112)	4.9% (48)	2.8% (27)	41% (404)	12% (113)	18% (175)	7.9% (77)	4.0% (39)	9.3% (91)	8.3% (81)	18% (173)	37% (358)	27% (263)	15% (143)	4.4% (43)	36% (355)	30% (291)	18% (177)	8.3% (81)	7.8% (76)
Other or prefer not to say	0% (0)	22% (2)	22% (2)	22% (2)	33% (3)	0% (0)	33% (3)	11% (1)	11% (1)	22% (2)	0% (0)	11% (1)	11% (1)	11% (1)	11% (1)	11% (1)	56% (5)	11% (1)	33% (3)	33% (3)	11% (1)	22% (2)	0% (0)
Ethnicity																							
White	28% (588)	35% (727)	20% (405)	11% (219)	3.8% (79)	2.8% (58)	44% (908)	10% (216)	15% (321)	8.1% (169)	4.0% (83)	9.4% (195)	8.9% (184)	17% (349)	39% (814)	25% (513)	15% (303)	4.7% (97)	35% (734)	28% (576)	18% (376)	10% (211)	8.6% (179)
Ethnic minority	30% (33)	38% (42)	15% (17)	8.1% (9)	6.3% (7)	2.7% (3)	48% (53)	14% (15)	18% (20)	3.6% (4)	4.5% (5)	4.5% (5)	8.1% (9)	24% (27)	39% (43)	19% (21)	15% (17)	2.7% (3)	37% (41)	37% (41)	14% (15)	5.4% (6)	7.2% (8)
Income																							
£26,000 or more	28% (466)	36% (602)	19% (318)	11% (174)	3.5% (58)	2.1% (35)	43% (716)	11% (178)	17% (274)	8.2% (135)	3.6% (59)	9.1% (151)	8.5% (140)	17% (275)	40% (668)	25% (411)	14% (231)	4.1% (68)	34% (569)	28% (470)	18% (305)	10% (170)	8.4% (139)
Less than £26,000	29% (157)	32% (172)	19% (105)	10% (55)	5.2% (28)	4.8% (26)	46% (249)	9.9% (54)	13% (70)	7.0% (38)	5.5% (30)	9.0% (49)	9.8% (53)	19% (103)	36% (193)	23% (125)	16% (89)	6.1% (33)	38% (207)	28% (151)	16% (88)	8.8% (48)	9.0% (49)
Social grade																							
ABC1	29% (481)	36% (588)	19% (319)	9.6% (158)	3.3% (54)	2.5% (41)	45% (731)	11% (174)	16% (261)	7.6% (125)	4.1% (67)	8.7% (142)	8.6% (141)	17% (280)	41% (669)	24% (391)	14% (229)	4.4% (72)	34% (553)	30% (492)	18% (298)	10% (170)	7.8% (128)
C2DE	26% (142)	34% (186)	19% (104)	13% (71)	5.8% (32)	3.6% (20)	42% (234)	10% (58)	15% (83)	8.6% (48)	4.0% (22)	10% (58)	9.4% (52)	18% (98)	35% (192)	26% (145)	16% (91)	5.2% (29)	40% (223)	23% (129)	17% (95)	8.6% (48)	11% (60)
Educational qualifications																							
Pre-16	34% (30)	28% (25)	15% (13)	12% (11)	10% (9)	1.1% (1)	43% (38)	9.0% (8)	21% (19)	1.1% (1)	10% (9)	11% (10)	4.5% (4)	18% (16)	44% (39)	17% (15)	16% (14)	5.6% (5)	40% (36)	27% (24)	13% (12)	11% (10)	7.9% (7)
Post-16	28% (593)	36% (749)	19% (410)	10% (218)	3.7% (77)	2.8% (60)	44% (927)	11% (224)	15% (325)	8.2% (172)	3.8% (80)	9.0% (190)	9.0% (189)	17% (362)	39% (822)	25% (521)	15% (306)	4.6% (96)	35% (740)	28% (597)	18% (381)	9.9% (208)	8.6% (181)
AUDIT category																							
8-15	37% (169)	46% (212)	12% (54)	5.0% (23)	0.6% (3)	0.2% (1)	32% (150)	7.8% (36)	18% (82)	5.2% (24)	7.1% (33)	11% (52)	18% (85)	37% (170)	50% (232)	8.7% (40)	3.2% (15)	1.1% (5)	32% (146)	32% (148)	19% (86)	9.5% (44)	8.2% (38)
16-19	35% (157)	41% (185)	16% (72)	6.6% (30)	1.3% (6)	0.4% (2)	38% (172)	10% (47)	17% (79)	7.7% (35)	5.1% (23)	11% (48)	11% (48)	22% (98)	49% (220)	22% (100)	6.6% (30)	0.9% (4)	34% (155)	29% (132)	17% (79)	10% (47)	8.6% (39)
20-40	23% (297)	29% (377)	23% (297)	14% (176)	6.0% (77)	4.5% (58)	50% (643)	12% (149)	14% (183)	8.9% (114)	2.6% (33)	7.8% (100)	4.7% (60)	8.6% (110)	32% (409)	31% (396)	21% (275)	7.2% (92)	37% (475)	27% (341)	18% (228)	9.9% (127)	8.7% (111)
Drinking affected by COVID-19																							
Yes	26% (277)	34% (367)	21% (220)	12% (125)	4.5% (48)	3.1% (33)	45% (486)	11% (117)	17% (180)	8.0% (86)	4.5% (48)	8.5% (91)	5.8% (62)	13% (138)	41% (437)	27% (289)	15% (160)	4.3% (46)	36% (387)	28% (302)	19% (202)	9.9% (106)	6.8% (73)
No	31% (346)	36% (407)	18% (203)	9.2% (104)	3.4% (38)	2.5% (28)	43% (479)	10% (115)	15% (164)	7.7% (87)	3.6% (41)	9.7% (109)	12% (131)	21% (240)	38% (424)	22% (247)	14% (160)	4.9% (55)	35% (389)	28% (319)	17% (191)	9.9% (112)	10% (115)
NA	0% (0)	0% (0)	0% (0)	0% (0)	0% (0)	0% (0)	0% (0)	0% (0)	0% (0)	0% (0)	0% (0)	0% (0)	0% (0)	0% (0)	0% (0)	0% (0)	0% (0)	0% (0)	0% (0)	0% (0)	0% (0)	0% (0)	0% (0)

Supplementary Table 8: Sensitivity analysis with complete case analysis for self-reported adherence, urges to drink, self-regulatory and self-monitoring behaviour as mediators of the intervention on alcohol reduction

	ACME (95% CI)	p-value	ADE (95% CI)	p-value
Self-reported adherence	-0.559 (-0.85 to -0.30)	<.001	-1.155 (-2.93 to 0.59)	.202
Self-monitoring behaviour	-0.880 (-1.24 to -0.55)	<.001	-0.835 (-2.57 to 0.99)	.390
Self-regulatory behaviour	0.169 (-0.47 to 0.80)	.612	-1.943 (-3.58 to -0.18)	.026
Urges to drink	-0.580 (-1.27 to 0.08)	.090	-1.238 (-2.94 to 0.39)	.122

Supplementary Table 9: Sensitivity analysis with complete case analysis for engagement measures as mediators of the effect of self-reported adherence on alcohol reduction in the intervention group

	ACME (95% CI)	p-value	ADE (95% CI)	p-value
Number of sessions	0.062 (-1.26 to 1.47)	.962	-7.76 (-11.57 to -3.75)	.002
Time on app	0.480 (-0.84 to 1.79)	.520	-8.24 (-11.68 to -4.65)	<.001
Number of days used	-0.101 (-1.36 to 1.23)	.880	-7.56 (-11.27 to -3.90)	<.001
Unique screens viewed	0.491 (-0.88 to 1.86)	.450	-8.16 (-12.17 to -4.24)	<.001
