# Supplementary material for: Do engagement and behavioural mechanisms underpin the effectiveness of the Drink Less app?
Source: NPJ Digit Med. 2024 Jun 29;7:174. doi: 10.1038/s41746-024-01169-7 (PMC11217434; doi:10.1038/s41746-024-01169-7)
Supplement: Supplementary file 2 — Reporting summary [file 41746_2024_1169_MOESM2_ESM.pdf]

nature portfolio

Corresponding author(s): *Double-anonymous peer review submissions: write DAPR and your manuscript number here instead of author names.*

Last updated by author(s): *YYYY-MM-DD*

Reporting Summary

Nature Portfolio wishes to improve the reproducibility of the work that we publish. This form provides structure for consistency and transparency in reporting. For further information on Nature Portfolio policies, see our Editorial Policies and the Editorial Policy Checklist.

Statistics

For all statistical analyses, confirm that the following items are present in the figure legend, table legend, main text, or Methods section.

n/a	Confirmed	
☐	☒	The exact sample size (*n*) for each experimental group/condition, given as a discrete number and unit of measurement
☐	☒	A statement on whether measurements were taken from distinct samples or whether the same sample was measured repeatedly
☐	☒	The statistical test(s) used AND whether they are one- or two-sided<br>*Only common tests should be described solely by name; describe more complex techniques in the Methods section.*
☐	☒	A description of all covariates tested
☐	☒	A description of any assumptions or corrections, such as tests of normality and adjustment for multiple comparisons
☐	☒	A full description of the statistical parameters including central tendency (e.g. means) or other basic estimates (e.g. regression coefficient) AND variation (e.g. standard deviation) or associated estimates of uncertainty (e.g. confidence intervals)
☐	☒	For null hypothesis testing, the test statistic (e.g. *F*, *t*, *r*) with confidence intervals, effect sizes, degrees of freedom and *P* value noted<br>*Give P values as exact values whenever suitable.*
☒	☐	For Bayesian analysis, information on the choice of priors and Markov chain Monte Carlo settings
☒	☐	For hierarchical and complex designs, identification of the appropriate level for tests and full reporting of outcomes
☒	☐	Estimates of effect sizes (e.g. Cohen's *d*, Pearson's *r*), indicating how they were calculated

Our web collection on statistics for biologists contains articles on many of the points above.

Software and code

Policy information about availability of computer code

Data collection	The anonymised data and data dictionary are available online at OSF (https://osf.io/2j9df). Microsoft Word and Excel are used.
Data analysis	The code for data analysis (R studio, v2023.06) is available online at OSF (https://osf.io/2j9df).

For manuscripts utilizing custom algorithms or software that are central to the research but not yet described in published literature, software must be made available to editors and reviewers. We strongly encourage code deposition in a community repository (e.g. GitHub). See the Nature Portfolio guidelines for submitting code & software for further information.

Data

Policy information about availability of data

All manuscripts must include a data availability statement. This statement should provide the following information, where applicable:

- Accession codes, unique identifiers, or web links for publicly available datasets
- A description of any restrictions on data availability
- For clinical datasets or third party data, please ensure that the statement adheres to our policy

The anonymised data, code and data dictionary are available online at OSF (https://osf.io/2j9df).

Research involving human participants, their data, or biological material

Policy information about studies with human participants or human data. See also policy information about sex, gender (identity/presentation), and sexual orientation and race, ethnicity and racism.

Reporting on sex and gender	Information has been collected on the gender of participants and descriptive statistics are reported stratified by gender.
Reporting on race, ethnicity, or other socially relevant groupings	Participants self-reported their ethnicity with the question: "What is your ethnic group?" with the following response options: white; black; Chinese; mixed; Asian; not known; other; prefer not to say.
Population characteristics	See 'research sample' below.
Recruitment	Participants were recruited between July 2020 and March 2022, and had to be aged 18+, increasing and higher risk drinkers (AUDIT score >=8), live in the UK, have access to an iOS device and want to drink less alcohol. Participants were recruited remotely via adverts on social media, the NHS website, radio adverts, and press releases and local advertising through health care providers.
Ethics oversight	University College London Research Ethics Committee (16799/001).

Note that full information on the approval of the study protocol must also be provided in the manuscript.

Field-specific reporting

Please select the one below that is the best fit for your research. If you are not sure, read the appropriate sections before making your selection.

☐ Life sciences ☒ Behavioural & social sciences ☐ Ecological, evolutionary & environmental sciences

For a reference copy of the document with all sections, see nature.com/documents/nr-reporting-summary-flat.pdf

Behavioural & social sciences study design

All studies must disclose on these points even when the disclosure is negative.

Study description	Quantitative data from a process evaluation of a randomised controlled trial.
Research sample	Participants were aged 18+, increasing and higher risk drinkers (AUDIT score >=8), live in the UK, have access to an iOS device and want to drink less alcohol. Just over half were women (57%) with a mean age of 42 years old, and the majority were white (95%).
Sampling strategy	We aimed to recruit a sample size of 5562 participants (2781 in the comparator group and 2781 in the intervention group) with over-recruitment by 50 people to account for possible removals due to duplicate responses or withdrawals, that were detected after data collection closed. This calculation was based on detecting a mean difference reduction of 2 UK units (16 g of alcohol) with an SD of 23 at 90% power with an alpha of 0.05 and a two-tailed test. The estimated effect size is in line with a Cochrane review on digital alcohol interventions and is roughly equivalent to that found in face-to-face brief intervention outcomes. The total sample recruited (after duplicate responses and withdrawals were removed) was 5,602, with n = 2814 in the comparator group and n = 2788 in the intervention group.
Data collection	Researchers were blind to study condition during data collection. Data were collected via Qualtrics (baseline and follow-up surveys) and via automatically collected data from within the app (intervention group only).
Timing	Participants were recruited from July 2020 to March 2022, and were then followed up for six months.
Data exclusions	No data were excluded from the analyses with multiple imputation used for any participants with missing 6-month follow-up data.
Non-participation	14,118 participants were assessed for eligibility, 7,300 did not meet inclusion criteria resulting in 6,818 being randomised. 1,206 of these were excluded due to fraudulent responses and 10 were excluded as the participant withdrew.
Randomization	Randomisation was generated by an online automated algorithm (at a ratio of 1:1), which tracked counts to ensure each intervention was displayed equally. Allocation was online and participants and researchers were masked to study arm.

Reporting for specific materials, systems and methods

We require information from authors about some types of materials, experimental systems and methods used in many studies. Here, indicate whether each material, system or method listed is relevant to your study. If you are not sure if a list item applies to your research, read the appropriate section before selecting a response.

Materials & experimental systems

n/a	Involved in the study
☒	☐ Antibodies
☒	☐ Eukaryotic cell lines
☒	☐ Palaeontology and archaeology
☒	☐ Animals and other organisms
☒	☐ Clinical data
☒	☐ Dual use research of concern
☒	☐ Plants

Methods

n/a	Involved in the study
☒	☐ ChIP-seq
☒	☐ Flow cytometry
☒	☐ MRI-based neuroimaging

Plants

Seed stocks	*Report on the source of all seed stocks or other plant material used. If applicable, state the seed stock centre and catalogue number. If plant specimens were collected from the field, describe the collection location, date and sampling procedures.*
Novel plant genotypes	*Describe the methods by which all novel plant genotypes were produced. This includes those generated by transgenic approaches, gene editing, chemical/radiation-based mutagenesis and hybridization. For transgenic lines, describe the transformation method, the number of independent lines analyzed and the generation upon which experiments were performed. For gene-edited lines, describe the editor used, the endogenous sequence targeted for editing, the targeting guide RNA sequence (if applicable) and how the editor was applied.*
Authentication	*Describe any authentication procedures for each seed stock used or novel genotype generated. Describe any experiments used to assess the effect of a mutation and, where applicable, how potential secondary effects (e.g. second site T-DNA insertions, mosiacism, off-target gene editing) were examined.*
